# Multimodal Ion Beam Imaging to Correlate Elements and Metabolites at the Micron Scale Using Water Cluster Secondary Ion Mass Spectrometry and MeV Ion Beam Analysis

**DOI:** 10.1021/acs.analchem.5c02890

**Published:** 2025-07-23

**Authors:** Catia Costa, Johanna von Gerichten, Vladimir Palitsin, Geoffrey W. Grime, Steve J. Hinder, Naoko Sano, Roger Webb, Melanie J. Bailey

**Affiliations:** † Ion Beam Centre, 3660 University of Surrey, Guildford GU2 7XH, U.K.; ‡ School of Chemical and Process Engineering, University of Surrey, Guildford GU2 7XH, U.K.; § The Surface Analysis Laboratory, University of Surrey, Guildford GU2 7XH, U.K.; ∥ Ionoptika, Southampton SO53 4BZ, U.K.; ⊥ Department of Infectious Diseases, Kings College London, London SE1 1UL, U.K.

## Abstract

Multiomics imaging at or below the single cell level is highly sought after for correlating the location of metal containing drugs, nanoparticles, or bioaccumulated metals with host metabolites and lipids. Secondary ion mass spectrometry (SIMS) is a technique that can image lipids and metabolites at high spatial resolution (∼1 μm), especially water cluster SIMS. Similarly, X-ray mapping techniques such as particle induced X-ray emission (PIXE) can image elements at submicron spatial resolution in tissues. Here we developed a workflow for SIMS followed by X-ray elemental mapping, performed on the same section of tissue. To enable compatibility with X-ray spectrometry, samples were mounted on a thin polymer film, which proved challenging for SIMS due to charge accumulation on the sample surface. Various sample preparation strategies, including carbon coating and metallic grids, were tested to overcome this issue. Multimodal imaging using SIMS and ion beam analysis (IBA) was then successfully performed on a porcine skin section. By way of example, we show how SIMS-IBA can be applied to image the different regions of a hair follicle to colocate elements, metals, and lipids using sequential elemental and molecular mapping, without any delocalization or loss by the preceding measurement.

## Graphical abstract

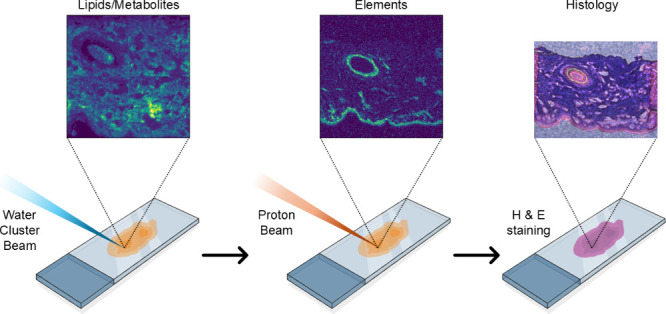

## Introduction

Correlating the location of elemental and molecular biomarkers is desirable for a wide range of applications in biology, including associating the location of metal nanoparticles, radioisotopes, or metal containing drugs with host metabolites to optimize their delivery. It is also important for understanding the biological pathways implicated in the bioaccumulation of metals.^1–6^

Ion beam analysis (IBA) techniques are a suite of techniques that can be very powerful for elucidating the subcellular location of metal particles. The techniques include particle induced X-ray (PIXE) and Rutherford backscattering (RBS) and use energetic primary ion beams (MeV energy range) to enable the accurate determination of the elemental composition of a sample, with analysis at submicron dimensions laterally and in depth. The techniques are quantitative in nature and have limited susceptibility to matrix effects.^2,7^ IBA techniques are widely used for the analysis of cells and tissues, for example to study metal dyshomeostasis related to Parkinson’s disease,^8^ copper depletion in atherosclerosis artery samples, or investigation of the uptake of metal-containing drugs into cells.^9^ However, a limitation of these techniques is that they do not carry any molecular information, therefore providing limited biological insight.

To address this problem, we have been exploring different strategies for performing correlative mass spectrometry imaging (MSI) with ion beam analysis.^2,10–13^ At single cell or subcellular scales, it is highly desirable to perform both analyses on the same sample, so that the same tissues or cells are analyzed by both techniques. We have recently shown that IBA techniques are compatible with the MSI approach desorption electrospray ionization (DESI), enabling sequential molecular and elemental imaging analysis on the same biological sample.^11–13^ While these studies have enabled correlative analysis of elements and host biomolecules at the tissue level, the techniques work at different length scales (routinely 1 μm for IBA versus typically 50 μm for DESI (best reported is 5 μm^14^)), limiting the ability to correlate organic and inorganic species at cellular and subcellular dimensions.

Secondary ion mass spectrometry (SIMS) is a mass spectrometry imaging technique that operates at spatial scales similar to MeV ion beam analysis,^15^ making it a suitable candidate for multimodal imaging. It has been used in various biological applications^16–18^ where determining the spatial distribution of biomolecules at the tissue (or even cellular) level has been used to optimize drug delivery and provide information on disease pathogenesis.^19,20^ Various primary ion sources (atomic, polyatomic, or cluster beams) are available for SIMS, with complementary features. Bismuth is a primary ion beam that provides excellent lateral resolution and the rare ability to detect both elemental and small molecular species in the same sample.^21^ However, for molecular species, bismuth SIMS carries some drawbacks, including extensive molecular fragmentation and matrix effects, which restricts peak identification and quantitation.^22^

A significant new development in SIMS is the use of gas cluster ion beam (GCIB) sources. GCIBs employing water clusters ((H_2_O)_*n*_^+^) show enhanced secondary ion yields for organic molecules compared to other primary ion clusters such as C_60_ and argon beams.^18,23–27^ The ability to ionize and detect intact molecular species such as amino acids, metabolites, and lipids makes water cluster SIMS an attractive tool for metabolomics imaging in cells and tissues.^24–26,28^

Here we explore the sequential use of IBA after bismuth and water cluster SIMS, with the ultimate aim of delivering submicrometer multimodal imaging. There are several challenges to overcome in the integration of techniques for the analysis of the same biological sample. The first is the substrate compatibility. To obtain good quality images and spectra, tissue samples are normally mounted on an electrically conductive substrate for SIMS (e.g., silicon wafer, metal). However, such substrates are incompatible with X-ray spectrometry, which typically uses polymer-based substrates (e.g., PET and polypropylene)^11–13^ that are thin and contain light elements (e.g., carbon, oxygen, and nitrogen). These polymer-based substrates are nonconductive, thereby creating a challenge for SIMS analysis.

The second challenge for integration is the impact of performing one technique upon another measurement as both techniques are at least partially destructive to the sample. We have previously shown that MeV ion beam analysis has a significant impact on the intensity of biomolecules detected by mass spectrometry imaging and similarly, mass spectrometry imaging can delocalize the trace elements that IBA techniques detect and image.^11,29^

In this work, we therefore have tested different sample handling arrangements for bismuth and water cluster SIMS. We also investigate whether SIMS leads to any loss or delocalization of elemental species by performing sequential IBA analysis on the same area of the same sample. We then demonstrate IBA-SIMS sequential imaging on a sample of porcine skin, showing how this combination of techniques can be used to correlate the distribution of elements such as Cl, S, P, K, and Zn with lipids.

This is, to the best of our knowledge, the first demonstration of sequential X-ray spectrometry and secondary ion mass spectrometry on the same tissue sample. This work should be of interest to researchers developing multimodal imaging approaches or those with an interest in colocating lipids and elements at the single or subcellular level in tissue.

## Materials and Methods

### Sample Preparation

Rat liver homogenates were prepared as described by Swales et al.^30^ Liver tissue was homogenized and pipetted into molds (2 mL bottom end of Pasteur pipet bulb). The homogenates were snap frozen in propanol and then isopentane and stored at −80 °C before cryosectioning to 12 μm thickness, unless otherwise stated. Full thickness porcine skin was sourced from Wetlab Medmeat supplies (Warwickshire, UK) and stored at −20 °C before use. Porcine skin was sectioned at the National Physical Laboratory (NPL, Teddington, UK) using a Leica CM 1850 Cryostat (Leica Microsystems, Wetzlar, Germany) at −20 °C. Sections were thaw-mounted onto substrates as required, lightly dried under argon gas, vacuum packed, and stored at −80 °C until analysis.

All animals and tissue were managed in accordance with the UK Home Office Animals (Scientific Procedures) Act 1986. The organs used in this study are within the 3Rs principles, as they comprise control material surplus to the original study for which they were intended.

### Sample Substrates

Various sample arrangements were tested for compatibility with both SIMS and IBA. Si wafers and indium tin oxide (ITO) slides (Diamond Coatings, Ltd., UK) were used as control substrates to benchmark the performance of the IBA-compatible substrates against substrates commonly used for SIMS and mass spectrometry imaging. These were compared against 1.4 μm PET and 4 μm PEN membrane slides (Leica, UK), chosen for potential compatibility with X-ray spectrometry. Carbon coating (estimated to be 10–20 nm thick) was achieved by using an Edwards E306 Auto Vacuum Coater with a carbon evaporation source.

The sample stage used for water cluster SIMS (IonOptika demo instrument) could not accommodate full-sized membrane slides, so custom-made sample holders were prepared. A smaller frame (25 × 14 mm) was made in-house and glued to PET or PEN membranes using super glue. The film was then cut around the smaller frame by using a scalpel and lifted off for analysis. Figure S1 shows the resulting sample arrangements used for water cluster SIMS analysis.

### SIMS Imaging

#### Bi-SIMS Imaging

Bi-SIMS analyses were carried out using an ION-TOF GmbH TOF.SIMS.5 instrument. A 25 keV Bi_3_^+^ primary ion beam delivering 1.0 μA of current was operated in the high current bunched mode. The MacroRaster mode was employed, where the stage moves under the beam to provide a mosaic of the total area analyzed. All data were acquired with a 100 μs TOF cycle. Unless stated otherwise, data was acquired in positive and negative ion modes. Table S1 shows the experimental parameters used for the Bi-SIMS measurements. All spectra and ion images were acquired by using “SurfaceLab 6.5”.

Data analysis was carried out using “SurfaceLab 7.3” provided by ION-TOF. For each sample, spectra were extracted from three regions of interest (ROIs). All spectra were calibrated using the peak list presented in Table S2. A list of biologically relevant fragment peaks in positive ion mode was constructed using the spectral library available in SurfaceLab (see Table S3) and checked against the acquired data, ensuring the peak is present in the spectrum and it originates from the tissue, not the substrate. Negative ion peak lists are reported in Table S4. All ion maps and extracted peak areas/intensities were normalized to the total ion count (TIC).

#### Water Cluster SIMS

Water cluster SIMS analysis was performed on a J105 SIMS (Ionoptika, Ltd., UK), as described previously.^31^ Two sets of optimization experiments were carried out using water cluster SIMS and the experimental parameters are described in Table S5. Data was acquired in positive and negative ion modes.

The first experiment used PET on custom frames as a substrate for liver tissue homogenates and explored different carbon coating arrangements: no carbon coating (PET), on top of the tissue sample (C-coated sample), and below the tissue sample (C-coated PET). A Si wafer was used as the control substrate. A porcine skin section (18 μm thickness) was mounted on a PET custom frame and carbon coated.

For the second experiment, liver tissue homogenates were mounted on custom frames with PET or PEN or in the presence of a metallic grid on top of the polymeric substrate, as shown in Figure S1. A homogenate section mounted on ITO was used as control. A porcine skin section (18 μm thickness) was mounted on a PEN substrate and a metallic grid was mounted on top. The grid, shown in Figure S1­(B), is made of aluminum and has a grid pitch of 2 × 2 mm. The total grid size is 2.5 × 2 cm.

Data was analyzed using Ionoptika Image Analyzer. Regions of interest (ROI) were drawn up to generate spectra from these regions and observe the variation of the peak intensities across a measurement. A list of lipids and respective fragments were matched against the spectral data (Table S4). All ion maps were normalized to the total ion image.

### Ion Beam Analysis

Following SIMS analysis, selected samples were analyzed simultaneously by particle induced X-ray emission (PIXE) and elastic backscattering spectrometry (EBS) using a 2 MV Tandem accelerator (High Voltage Engineering, Netherlands). Samples were placed in a vacuum chamber pumped to 10^–6^ mBar and irradiated using 2.5 MeV protons with beam currents ranging 300–600 pA. The beam was focused to approximately 1–3 μm (measured using a 75 × 75 μm 1000 copper grid). The pixel dwell time was set to 0.3 ms and a resolution of 256 pixels × 256 pixels. X-rays were detected using a silicon drift detector (SDD) fitted with a 130 μm Be filter mounted at an angle of 135° to the beam direction in the horizontal plane. Backscattered particles were simultaneously collected and detected using a PIPS detector with an active area of 150 mm^2^, placed 52.5 mm away from the sample and mounted at a 25° exit angle. Samples were analyzed until a charge of 1000 or 4000 nC/mm^2^ was reached for liver homogenates and porcine skin sections, respectively.

The X-ray and backscattered particle spectra were calibrated by using a BCR-126A lead glass standard. Data were acquired and analyzed using OMDAQ-3 software (Oxford Microbeams, Ltd., UK).^32^ All PIXE maps were normalized to the total RBS map to account for changes in sample thickness and density.

### Statistical Analysis

Most of the positive ion peaks being used for comparison in this data set are fragments that can originate from several different parent ions, causing a lack of independent features and too much multicollinearity for most statistical tests to handle. Normality tests (D-Agostino & Pearson, Anderson–Darling, Shapiro–Wilk, and Kolmogorov–Smirnov tests) were carried out on each data set. Based on the results, Kruskal–Wallis with Dunn’s multiple comparisons test, or ordinary one-way ANOVA were carried out to compare the different sample arrangements for each experiment. Where appropriate, single or multiple *t* tests were also carried out.

## Results and Discussion

### PET Membranes Are Compatible with SIMS Imaging

The conductive and pure nature of silicon wafers make them an ideal substrate for SIMS imaging of tissues.^33–35^ However, as reported by de Jesus et al.,^13^ silicon is not appropriate for PIXE and other X-ray spectrometry techniques, which require a substrate of low Z to avoid interferences. In contrast, PET substrates are compatible with X-ray spectrometry, as well as other modalities such as histology and laser capture microdissection.^36^ We therefore tested the compatibility of SIMS imaging with tissues mounted on PET substrates. Due to the insulating nature of the PET substrate, we also tested carbon coated PET (C-PET). The performance was benchmarked against that of a silicon substrate. The sample arrangements are presented in Figure 1(B).

**Figure 1:**
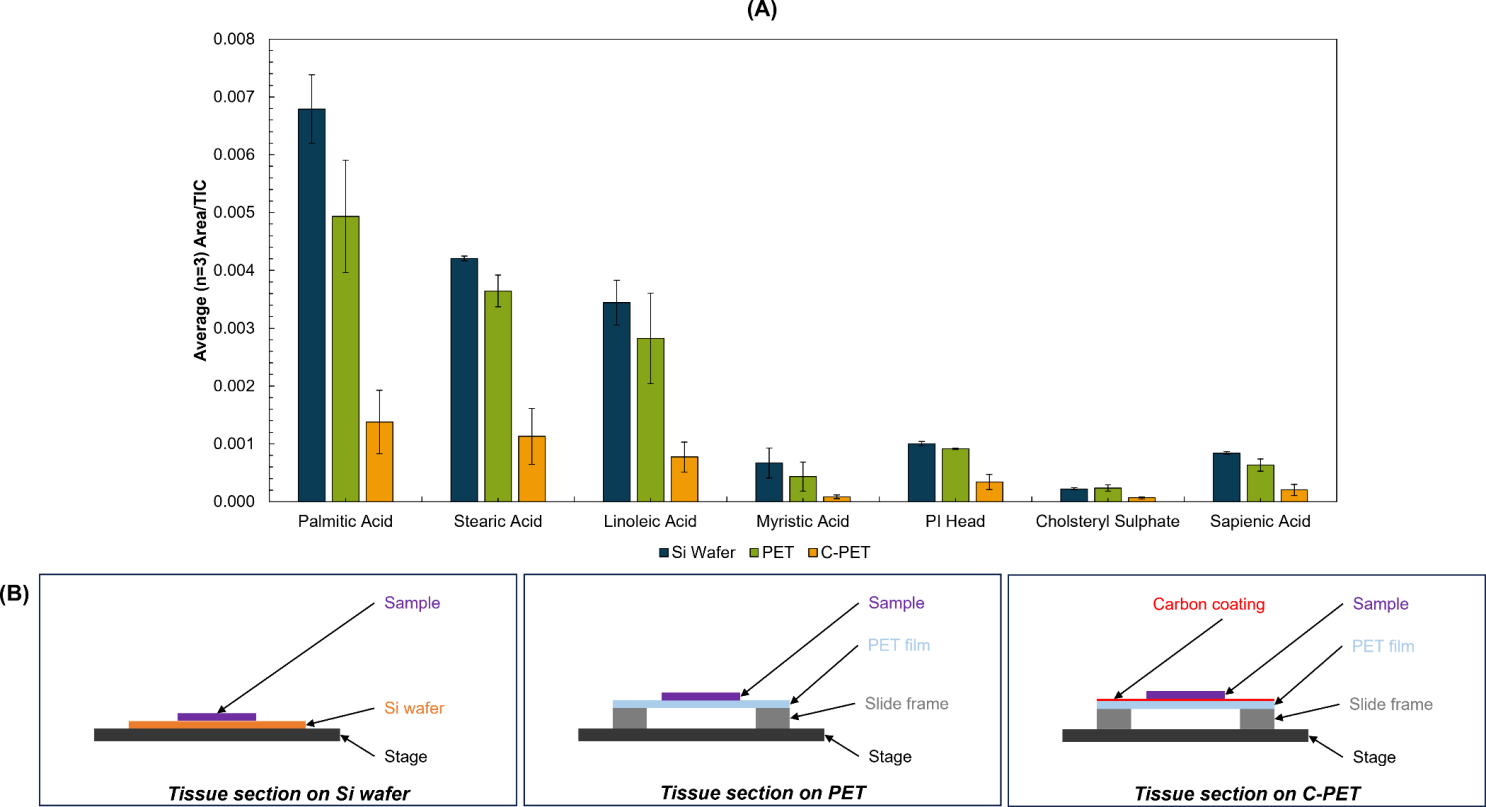
(A) Average (*n* = 3) TIC normalized peak area of tentatively assigned features in negative ion mode measured using bismuth SIMS from liver tissue homogenates mounted on Si wafer (blue), PET (green), and carbon coated PET (C-PET; yellow) substrates. (B) Schematic representation (not to scale) of the cross-section view of the sample arrangements described for this experiment.

Figure 1(A) shows how the TIC-normalized intensity of a selection of tentatively identified analytes from the tissue detected in negative ion mode depends on the substrate used. For the analytes monitored in negative mode, PET and Si wafer provide similar intensities, while the use of C-PET led to lower peak intensities (confirmed by multiple *t* tests, Table S6). In positive ion mode, no significant difference was observed between the three sample arrangements (Figure S2 and Table S7). A comparison of ion maps generated from each substrate is presented in Figure S3, showing lower contrast between on and off tissue regions when C-PET is used. This is particularly obvious for negative ion mode maps. As shown in Figure 1(B), the carbon coating was applied to the PET before thaw-mounting the tissue to (1) avoid aging effects on the tissue and (2) avoid introducing potential contaminants during coating. However, based on Figure 1A, it would appear that placing the carbon layer beneath the sample reduces sensitivity. PET was, therefore, used for subsequent experiments.

In previous multimodal imaging work,^11–13^ the tissue section was placed on a PET membrane as illustrated in Figure 1, defined as a top mounted configuration. In this arrangement, there is a space (a few millimeters) between the PET film and the stage. While this configuration was suitable for DESI imaging, we noted that this orientation presented some difficulties in charge compensation during the setup of the SIMS measurements. Charge could not be fully compensated, and the reflectron voltage had to be set to −500 V, the maximum allowed by the software/hardware.

To optimize the mounting orientation for PET, a sequential section of tissue was mounted on a PET membrane flipped by 180°, defined as reverse mounted, illustrated in Figure 2. This configuration gives direct contact between the PET membrane and the stage, allowing for better charge compensation. The average TIC-normalized peak areas determined for the two sample arrangements are presented in Figure S4, showing that the sample configuration has limited effect on peak area. However, Figure 2 shows images constructed from a selection of peaks detected in positive mode using the two sample arrangements, clearly showing that the reverse mounted configuration provides sharper images.

**Figure 2:**
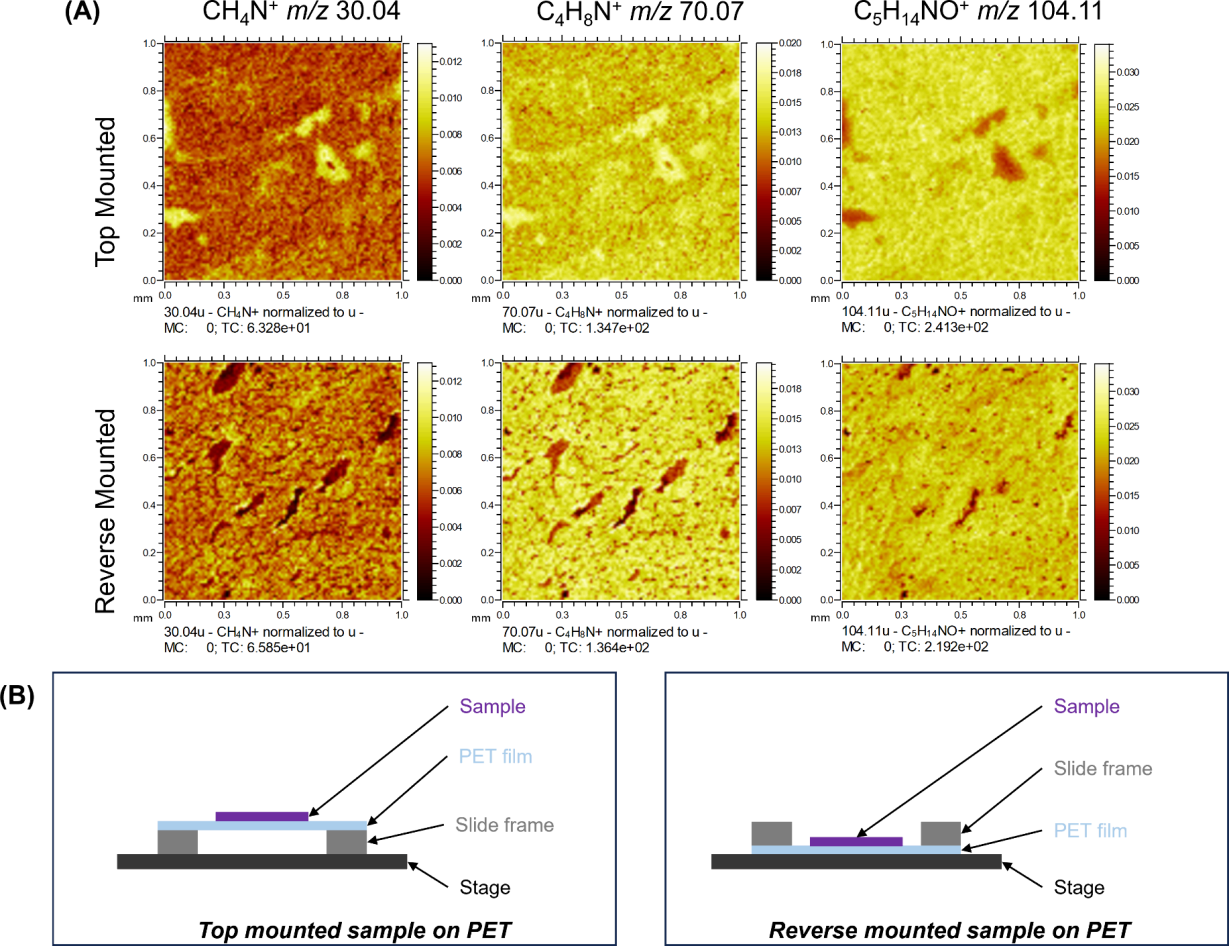
(A) Bi-SIMS ion maps (1 mm × 1 mm) measured from Area 1 on liver tissue homogenates top and reverse mounted on PET membrane slides and (B) schematic representation (not to scale) of the cross-sectional view of the sample arrangements described for this experiment.

A liver tissue homogenate sample mounted on PET was sequentially analyzed using Bi-SIMS followed by IBA to investigate whether the bismuth primary ion beam caused any changes to the sample. Figure S5 shows the resulting PIXE and EBS maps, which clearly show there is no observable delocalization of elements after Bi-SIMS analysis. These results therefore provide proof of principle that SIMS can be carried out on a PET membrane, prior to elemental mapping.

### The Sample Handling Requirements for Water Cluster SIMS and Bi-SIMS Are Different

A liver homogenate sample was prepared using the optimal arrangement determined for Bi-SIMS, specifically, a tissue section on a PET slide in the reverse mounted configuration. However, unlike Bi-SIMS, reverse mounted PET membranes gave poor signal intensity when compared with silicon substrates and poor-quality images. This can be explained by differences in the nature and mode of operation of the primary ion beams used. First, water molecules are polar, and it has been shown recently^37^ that water can generate charge when it moves across a surface and therefore the charging effects on insulating substrates can be expected to be inherently greater than with bismuth. Second, the Bi- SIMS uses a pulsed primary ion beam, in contrast to the continuous primary ion beam in the water cluster SIMS system, and therefore the input of charge is higher. There are also differences in the methodologies for charge compensation among the two systems. We therefore explored carbon coating strategies for water cluster SIMS, including carbon coating the PET before sectioning (C-coated PET), as well as carbon coating the sample after sectioning (C-coated sample). As shown in Figure 3 (and Figure S6 for an expanded set of features), both modes of carbon coating significantly improved the signal intensity. Carbon coating the sample led to higher yields of negative ions than carbon coating the PET only (see Tables S8 and S9 for statistical tests).

**Figure 3:**
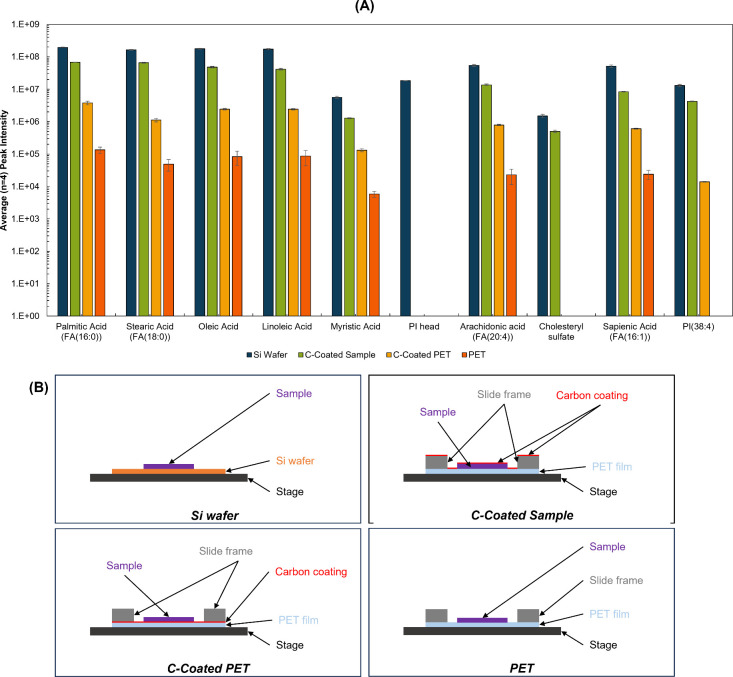
(A) Average (*n* = 4) TIC-normalized peak intensity of lipids measured using water cluster SIMS from liver tissue homogenates mounted on Si wafer and PET membrane slides in negative ion mode. (B) Schematic representation (not to scale) of the cross-section view of the sample arrangements described for this experiment.

### Sample Handling Requirements Can Be Sample Dependent

A porcine skin section was prepared and analyzed in accordance with the C-coated sample arrangement, determined in Figure 3 to give the closest intensity to silicon for tissue homogenates. However, Figure S7 shows the positive and negative ion spectra collected from porcine skin sample. There is a clear pattern of repeating units of 18 *m*/*z*, which can be explained by the detection of backscattered water clusters from the primary ion beam. This potentially arises from poor charge compensation, supported by the observation that the effect was most pronounced for the positive ion images. The differences observed between the liver homogenates and porcine skin can be explained by sample size and location with respect to the metal frame. It was noted that the distance between the measurement location and the metal frame (which helps dissipate any charge) was greatest for the porcine skin than the liver homogenates.

We therefore trialled a further set of sample preparation arrangements, aimed at mitigating charging effects and associated spectral artifacts. We tested PEN membranes due to their superior thermal and mechanical properties compared with PET and tested the use of grids placed above the sample as an alternative to carbon coating. These were benchmarked against tissue mounted on an ITO slide. Figure 4 and Figure S4 show the average (*n* = 3) intensities for a selection of lipid peaks in negative and positive ion mode, respectively. As shown in Figure 4, the addition of a metallic grid to either PET or PEN resulted in higher peak intensities. In fact, in the presence of a metallic grid, PEN and PET were found to perform equally well and similarly to ITO in either negative (Table S10) or positive (Table S11) ion modes.

**Figure 4:**
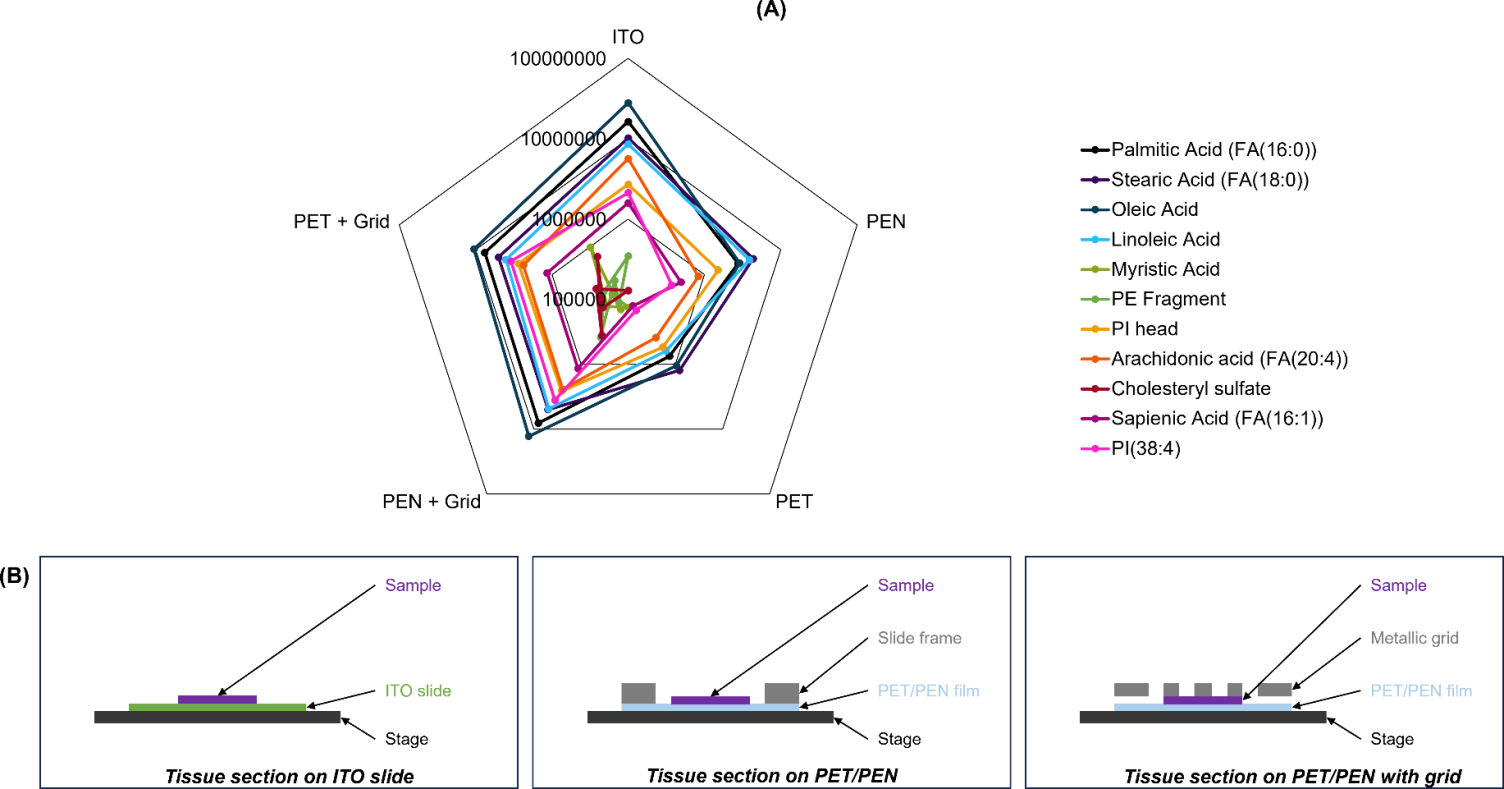
(A) Average (*n* = 3) peak intensity of tentatively assigned lipid peaks measured using water cluster SIMS from liver tissue homogenates prepared under different arrangements in negative ion mode. (B) Schematic representation (not to scale) of the cross-section view of the sample arrangements described for this experiment.

As such, PEN + Grid was used for a subsequent multimodal imaging demonstration because it yielded good sensitivity and was found to be easier to handle. It also carries the advantage of better heat resistance compared to PET and is compatible with laser capture microdissection.^38^

### Water Cluster SIMS Does Not Delocalize Elements

To test whether water cluster SIMS measurements caused any delocalization or loss of elements, porcine skin areas were analyzed with water cluster SIMS and the imaged areas were relocated and imaged using PIXE, as shown in Figure 5. The area highlighted by the green box shows the approximate location of the water cluster SIMS measurements. Inspection of these images inside and outside the green box shows that there is no observable loss or delocalization of elements in this region after the initial SIMS imaging. Given that the pixel size of these images is approximately 4 μm and the diameter of a mammalian cell is around 20 μm, we propose that these two techniques can be used sequentially in this order for tissue level analysis at the single cell level. To further confirm this, eight regions of interest (ROI) were drawn of the top left and middle left squares of the mosaic (as shown in Figure S9), and the concentrations of phosphorus (P), sulfur (S), chlorine (Cl), and potassium (K) were calculated for each of the ROIs. These two areas were selected as they encompass areas analyzed by SIMS (top left) and not analyzed by SIMS (middle left). The graphical insert in Figure 5 shows the calculated concentrations normalized to the number of pixels in each ROI, giving no significant difference (*p* > 0.05) in terms of their elemental concentrations (Table S12).

**Figure 5:**
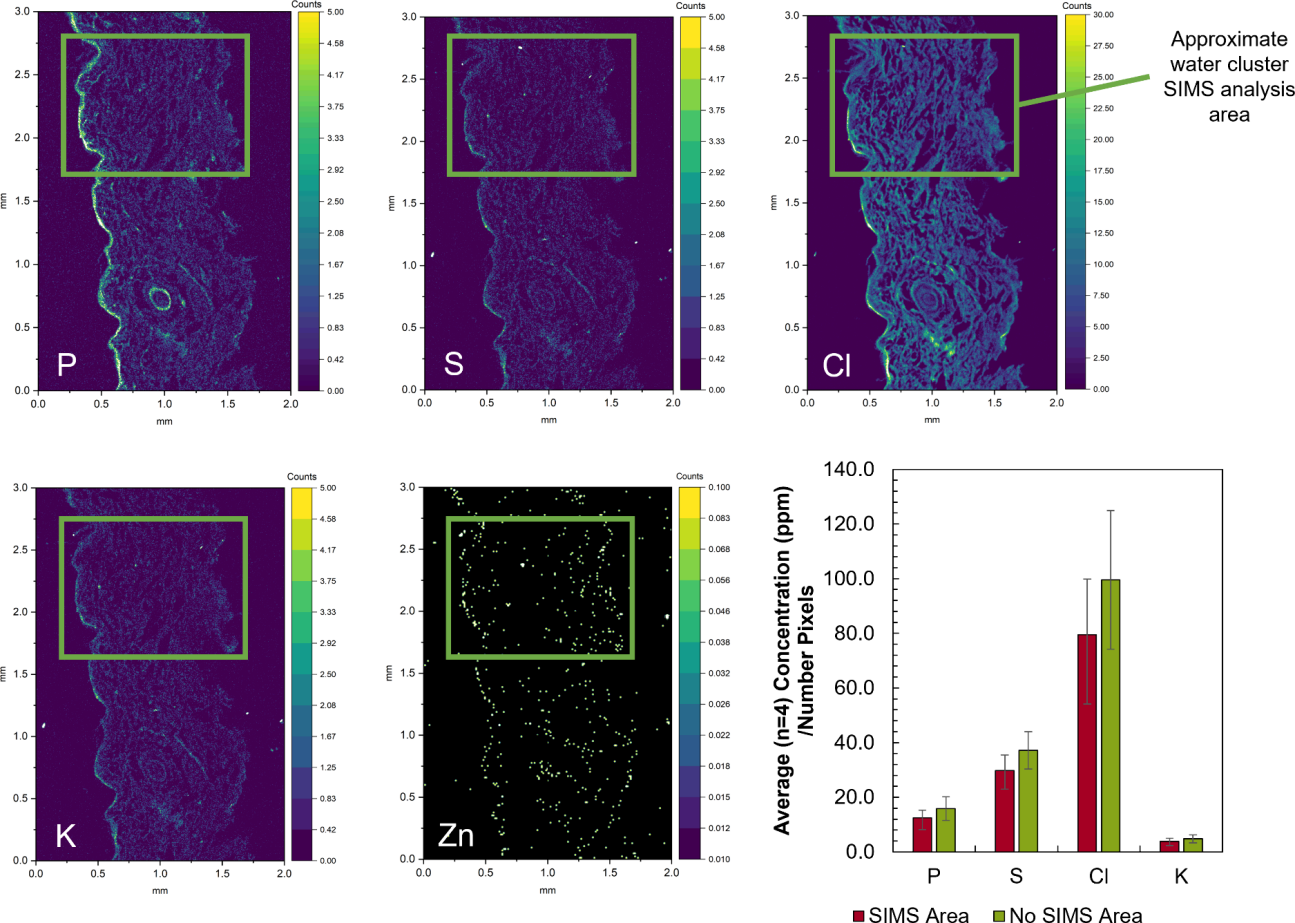
PIXE maps for phosphorus (P), sulfur (S), chlorine (Cl), potassium (K), and zinc (Zn) acquired after water cluster SIMS analysis in region marked by green square. Calculated concentrations (in ppm; normalized to the number of pixels in the ROI) measured for phosphorus (P), sulfur (S), chlorine (Cl), and potassium (K) for areas with/without prior SIMS analysis. Images are a mosaic of 1 mm squares, in a 2 × 3 arrangement.

### Demonstration of Multimodal Imaging of Porcine Skin Using Water Cluster SIMS and Ion Beam Analysis

To demonstrate multimodal imaging, a further porcine skin section was prepared in the PEN + Grid configuration and analyzed using sequential water cluster SIMS and PIXE. The sample was then submitted to H&E staining to enable annotation of the difference anatomical regions. These were identified with reference to literature^39,40^ and clearly show the hair shaft, inner root sheath (IRS), and outer root sheath (ORS) (Figure 6 and Figure S10). The anatomy of the hair follicle, and of the tissue in general, is still discernible, demonstrating that H&E staining is possible after multimodal ion beam imaging.

**Figure 6:**
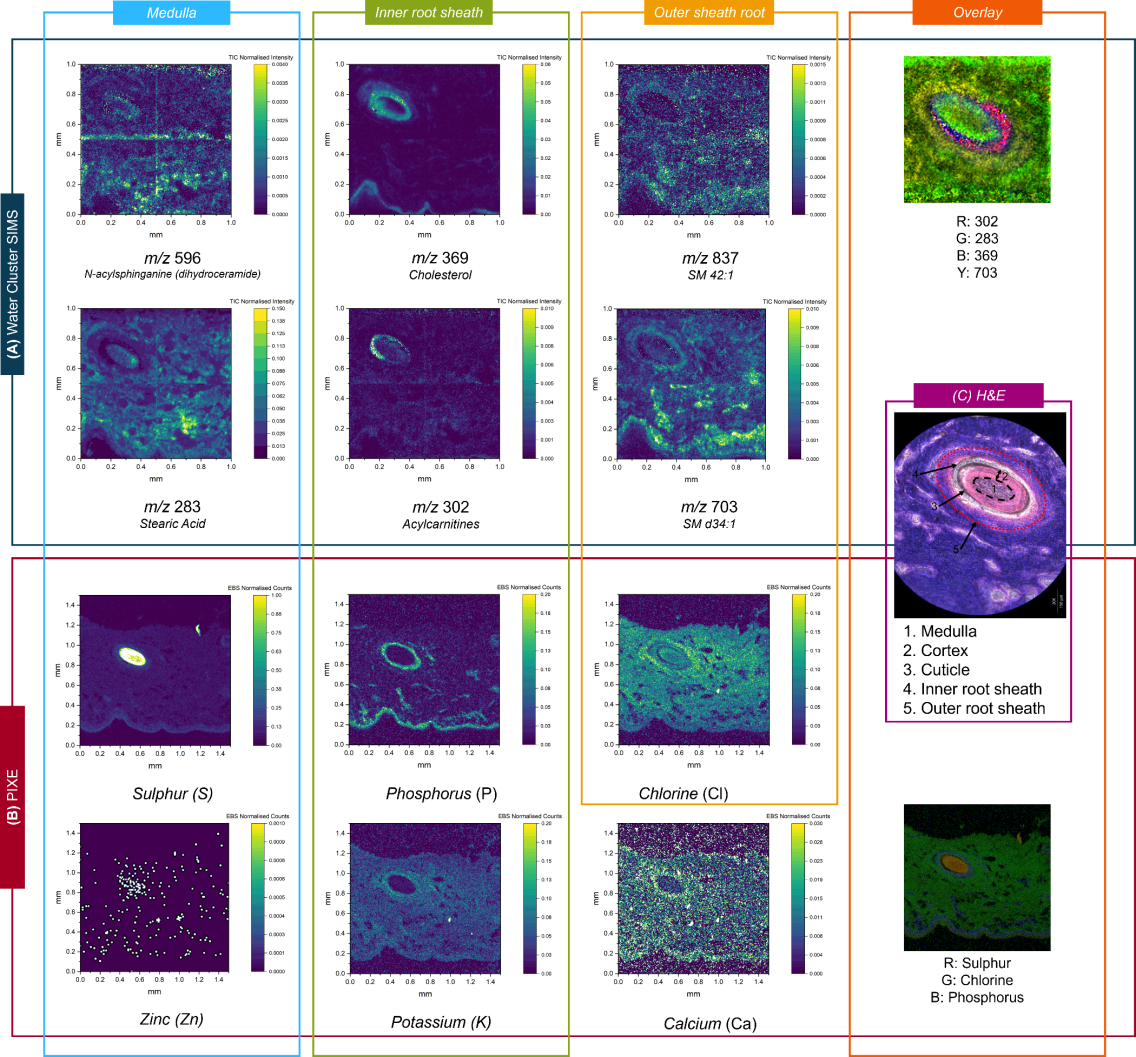
Water cluster SIMS (A) and PIXE (B) maps taken from a porcine skin sample mounted on PEN with a metallic grid on top. (C) H&E stained optical image taken after water cluster SIMS and PIXE.

The different regions of the hair follicle identified through H&E staining can be used to identify anatomical regions in the water cluster SIMS images, and these are validated using *m*/*z* peaks previously identified in the literature.^39^ Specifically, the medulla is characterized by the presence of *m*/*z* 596, assigned to *N*-acylsphinganine (dihydroceramide); the inner root sheath is characterized by *m*/*z* 369 (cholesterol) and the outer root sheath by *m*/*z* 837 (SM 42:1).^39,40^ Other peaks originating from each of these areas included *m*/*z* 283 (in the medulla, assigned to stearic acid), *m*/*z* 302 (in the IRS, assigned to acetylcarnitines), and *m*/*z* 703 (in the ORS, assigned to SM d34:1). Figure 6 also shows the overlay of the different metabolites and elements originating from the different regions of the hair follicle, showing the structural organization of the hair follicle. This is the first demonstration of porcine skin analysis using water cluster SIMS.

Figure 6(B) also shows elements detected by PIXE, performed on the same section of tissue after water cluster SIMS imaging, and before H&E. Using the H&E to assign anatomical regions, the medulla region is characterized by elevated levels of sulfur and zinc. Sulfur is an important component of keratin, the protein that makes up hair. Zinc supports the production of keratinocytes, the cells that produce keratin.^41^ The PIXE images show that the inner root sheath is characterized by elevated levels of phosphorus. The SIMS images corroborate this, showing elevated phosphorus-containing lipids (as shown in Figure S11 by the distribution of the PC and PI headgroups) in this region. The PIXE images in Figure 6(B) show that this region is also characterized by elevated Ca^42^ and K,^43^ thought to play an essential role in keratinocyte differentiation. Similarly, cholesterol sulfate plays an important role in keratinocyte differentiation by forming a protective barrier “integral hair lipid” around the hair shaft, which helps maintain hair health.^40,44^ The water cluster SIMS maps shown in Figure S12 for *m*/*z* 465 (negative ion mode) show evidence of cholesterol sulfate colocated with Ca and K in this region, and the peak assignment is further supported by the detection of cholesterol (*m*/*z* 369) and cholesterol oleate (*m*/*z* 673), also shown in Figure S11.^45^

It is known that sphingolipids help maintain the barrier function of the skin by forming a protective layer around the hair follicle in the outer root sheath,^46^ preventing excessive water loss and protecting against external stressors. This is supported by the detection of *m*/*z* 837 and 703 in the outer root sheath by water cluster SIMS, assigned to sphingolipids. The PIXE images show that chlorine (Cl) is also upregulated in the outer root sheath region. While we cannot find other reports of Cl distribution in hair follicles, it is possible that the outer root sheath accumulates Cl to protect the hair from excess Cl salinity,^47^ as reported in maize.

We have developed a workflow that can be applied to biological tissues for multimodal elemental and molecular imaging using keV and MeV ion beams on the same sample. The data presented here demonstrate that water cluster SIMS does not cause loss or delocalization of elemental species, as measured by MeV ion beam analysis. In addition, our data implies that other elemental mapping techniques, for example, other X-ray spectrometry approaches, can be applied to the same tissue sample following SIMS analysis. For example, it may be possible in future work to use this approach to explore the impact of metal accumulation, metal nanoparticles, and metal-containing drugs (detected by elemental mapping) on the host metabolome (detected by SIMS). It also opens the possibility of using multimodal MeV and keV ion beams to gain a better understanding of matrix effects in SIMS imaging since the MeV beam is blind to the sample chemistry.

We acknowledge that the information depth of PIXE and SIMS is different—the information from PIXE will come from the whole depth of these thin tissue samples, whereas the SIMS images come from only the top layer. At the single cell level, this is not necessarily a concern, since the same cells are being probed. However, this work opens up the exciting prospect of colocalizing metals or other elements with metabolites and lipids in biological systems below the single cell level, and future work will need to explore how to deal with the difference in information depths.

## Conclusions

The data presented here show how SIMS can be performed prior to elemental mapping by other imaging modalities. We have demonstrated that SIMS analysis can be performed on tissue sections mounted on polymer membranes to obtain images of biologically relevant species, opening up the possibility of performing X-ray spectrometry post-SIMS. We recommend mounting the tissue in a way that allows direct contact between the film and the metallic sample stage to aid charge transfer and the use of a metallic grid to further aid charge dissipation. Sequential analysis using water cluster SIMS for imaging organic molecules followed by PIXE/EBS for elemental mapping was successfully performed and showed that the preceding water cluster-SIMS analysis caused no delocalization or loss of elemental information. This presents the exciting prospect of colocalizing metals or other elements with lipids and metabolites at the single or, in future work, even subcellular level.

## Supplementary Material



## Notes

Experimental parameters; photos of sample arrangement; list of peaks for mass calibration and peak identification; statistical test results; supporting data in the form of graphs and maps; example spectra; optical images of samples after staining (PDF)
