# Supplementary material for: Multimodal Ion Beam Imaging to Correlate Elements and Metabolites at the Micron Scale Using Water Cluster Secondary Ion Mass Spectrometry and MeV Ion Beam Analysis
Source: Anal Chem. 2025 Jul 23;97(30):16573–82. doi: 10.1021/acs.analchem.5c02890 (PMC12332816; doi:10.1021/acs.analchem.5c02890)
Supplement: Supplementary file 1 [file ac5c02890_si_001.pdf]

Supporting Information

Multimodal ion beam imaging to correlate elements and metabolites at the micron scale using water cluster secondary ion mass spectrometry and MeV ion beam analysis

Catia Costa[a,§], Johanna von Gerichten[b, §], Vladimir Palitsin[a], Geoffrey W. Grime[a], Steve J. Hinder[c], Naoko Sano[d], Roger Webb[a] and Melanie J. Bailey[a,e*]

[§] C.C. and J.v.G. contributed equally to this work

[a]Ion Beam Centre, University of Surrey, Guildford, GU2 7XH, UK
[b]School of Chemical and Process Engineering, University of Surrey, Guildford, GU2 7XH, UK;
[c]The Surface Analysis Laboratory, University of Surrey, Guildford, GU2 7XH, UK
[d]Ionoptika, Southampton, SO53 4BZ, UK
[e] Department of Infectious Diseases, Kings College London, London, SE1 1UL, UK

* Email: melanie.j.bailey@kcl.ac.uk

List of Contents

Table S 1. 25 keV Bi_3^+ SIMS experimental parameters.

Table S 2. List of peaks used for calibration of the Bi-SIMS spectral data in positive and negative ion modes.

Table S 3. List of peaks extracted from SurfaceLab Spectral Library and respective accurate mass in positive ion mode. Orange colour = peak not detected; Green colour = Peak detected.

Table S 4. Calculated monoisotopic mass for lipids detected using SIMS.

Table S 5. Water cluster SIMS experimental parameters.

Table S 6. Multiple t-test results comparing the TIC normalised area measured from liver tissue homogenates mounted on Si wafer, PET and carbon coated PET (C-PET) and measured using Bi-SIMS in negative ion mode.

Table S 7. Kruskal-Wallis test results comparing Si wafer, PET and carbon coated PET (C-PET) as sample substrates in positive ion mode for Bi-SIMS.

Table S 8. Kruskal-Wallis test results comparing different samples arrangements (Si wafer, C-coated sample, C-coated PET and PET) tested on liver homogenates using water cluster SIMS in positive ion mode.

Table S 9. Multiple t-test results comparing the peak intensities measured from liver tissue homogenates mounted on Si wafer, PET and carbon coated PET (C-PET) using water cluster SIMS.

Table S 10. Mann Whitney tests results carried out between PEN + Grid and ITO, PET + Grid and ITO and PEN + Grid and PET + Grid datasets obtained from liver tissue homogenates and measured using water cluster SIMS in negative ion mode.

Table S 11. Kruskal-Wallis test results comparing different samples arrangements (ITO, PEN, PET, PEN + Grid and PET + Grid) tested on liver homogenates using water cluster SIMS in positive ion mode.

Table S 12. Multiple t-test results comparing the elemental concentrations measured using PIXE from an area that had previously been analysed with SIMS and a pristine (no SIMS) area.

Figure S 1. Sample holders used for water cluster SIMS analysis on Ionoptika's J105 instrument; (A) small frame and (B) metallic grid sample arrangements.

Figure S 2. (A-B) Average (n=3) TIC normalised peak area of tentatively assigned features in negative ion mode measured using bismuth SIMS from liver tissue homogenates mounted on Si wafer, PET and carbon coated PET (C-PET) substrates; (B) Schematic representation (not to scale) of the cross-section view of the sample arrangements described for this experiment.

Figure S 3. (A) Bi-SIMS ion maps (3 x 3 mm) measured from liver tissue homogenates mounted on Si wafer (left), PET (middle) and C-PET (right); (B) Schematic representation (not to scale) of the cross-section view of the sample arrangements described for this experiment.

Figure S 4. (A-B) Average (n=3) TIC-normalised peak area of tentatively assigned fragment peaks in positive ion mode measured using Bi-SIMS from liver tissue homogenates measured from top and reverse mounted arrangements; (C) Bi-SIMS ion maps (1 x 1 mm) measured from Areas 1, 2 and 3 on liver tissue homogenates top and reverse mounted on PET membrane slides and (D) schematic representation (not to scale) of the cross-section view of the sample arrangements described for this experiment.

Figure S 5. PIXE and EBS maps of liver tissue homogenates mounted on PET analysed sequentially using Bi-SIMS and ion beam analysis. Images are 256 x 256 pixels in a 1 x 3 array.

Figure S 6. (A) Average (n=4) TIC-normalised peak intensity of tentatively assigned lipid peaks measured using water cluster SIMS from liver tissue homogenates prepared under different arrangements positive ion mode.

Figure S 7. Total ion spectra for (A) positive and (B) negative ion modes showing the repeating 18 Da units originating from the backscattered water clusters; (C) ion maps for a selection of lipid-related peaks obtained from a carbon-coated porcine skin section, mounted on a PET membrane and analysed using water cluster SIMS.

Figure S 8. (A) Average (n=3) peak intensity of tentatively assigned lipid peaks measured using water cluster SIMS from liver tissue homogenates prepared under different arrangements in positive ion mode; (B) schematic representation (not to scale) of the cross-section view of the sample arrangements described for this experiment.

Figure S 9. Regions of interest (ROI) selected from an area previously analysed by water cluster SIMS and a pristine (no previous measurement) area.

Figure S 10. Porcine skin sample mounted on PEN with a metallic grid on top (as shown in Figure S1(B)). H&E staining was performed after sequential water cluster SIMS and IBA. Optical images taken at (A) 4x, (B) 10x and (C) 20 x magnification. (C) also shows the annotated regions of the hair follicle.

Figure S 11. Ion maps taken from a porcine skin sample mounted on PEN with a metallic grid on top and analysed using water cluster SIMS in positive (top row) and negative (bottom row) ion modes.

Figure S 12. Ion maps for cholesterol-derived peaks taken from a porcine skin sample mounted on PEN with a metallic grid on top and analysed using water cluster SIMS.

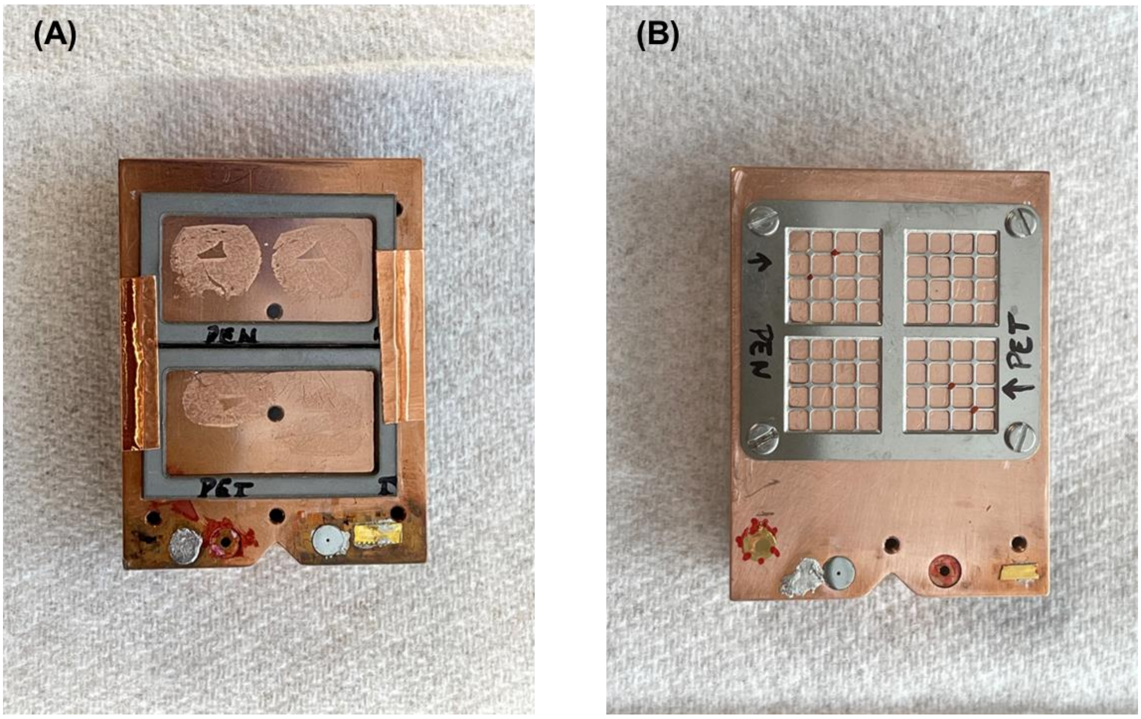

Figure S 1. Sample holders used for water cluster SIMS analysis on Ionoptika's J105 instrument; (A) small frame and (B) metallic grid sample arrangements.

Table S 1. 25 keV Bi_3^+ SIMS experimental parameters.

	No. of scans	Shots/pixel	Pixel resolution	Frames/patch	Maximum patch length (mm)	Pixel density (pixel/mm)	Total analysis area (µm)
**Exp. 1**	1	10	128x128	1	0.25	100	1000
**Exp. 2**	1	50	128x128	1	0.25	100	1000

Table S 2. List of peaks used for calibration of the Bi-SIMS spectral data in positive and negative ion modes.

Assignment	Accurate *m/z*		Assignment	Accurate *m/z*
Positive Ions				
H^+	1.0073		$C_4H_9^+$	57.0699
CH_2^+	14.0151		$C_4H_{10}^+$	58.0777
CH_3^+	15.0229		$C_5H_5^+$	65.0386
NH_4^+	18.0333		$C_5H_6^+$	66.0464
OH_3^+	19.0178		$C_5H_7^+$	67.0542
Na^+	22.9892		$C_5H_9^+$	69.0699
Mg^+	23.9845		$C_6H_6^+$	78.0464
$C_2H_2^+$	26.0151		$C_6H_7^+$	79.0542
$C_2H_3^+$	27.0229		$C_6H_8^+$	80.0621
$C_2H_4^+$	28.0308		$C_6H_9^+$	81.0699
$C_2H_5^+$	29.0386		$C_6H_{11}^+$	83.0855
CH_3O^+	31.0178		$C_6H_{12}^+$	84.0934
K^+	38.9632		$C_6H_{13}^+$	85.1012
$C_3H_5^+$	41.0386		$C_6H_{14}^+$	86.1090
$C_3H_7^+$	43.0542		$C_7H_8^+$	92.0621
$C_4H_2^+$	50.0510		$C_7H_{11}^+$	95.0855
$C_4H_3^+$	51.0229		$C_7H_{13}^+$	97.1012
$C_4H_4^+$	52.0308		$C_5H_{14}NO^+$	104.1070
$C_4H_5^+$	53.0386		$C_2H_6PO_4^+$	124.9998
$C_4H_6^+$	54.0464		$C_5H_{13}PNO_3^+$	166.0628
$C_4H_7^+$	55.0542		$C_5H_{15}PNO_4^+$	184.0733
Negative Ions				
H^-	1.008		CHO^-	29.003
C^-	12.000		CH_3O^-	31.019
CH^-	13.008		S^-	31.973
CH_2^-	14.016		Cl^-	34.969
O^-	15.995		C_3^-	36.001
OH^-	17.003		$^{37}Cl^-$	36.967
C_2^-	24.001		$C_3H_2^-$	38.016
C_2H^-	25.008		$C_4H_3^-$	51.024
CN^-	26.004		PO_3^-	78.959
$C_2H_3^-$	27.024			

Table S 3. List of peaks extracted from SurfaceLab Spectral Library and respective accurate mass in positive ion mode. Orange colour = peak not detected; Green colour = Peak detected.

	**Required Peaks**		**Characteristic Peaks**	
	Assignment	*Accurate Mass*	*Assignment*	*Accurate Mass*
**Ceramide 36:1**	$C_{36}H_{72}NO_3^+$	567.56	$C_{18}H_{32}O^+$	264.24
	$C_{36}H_{71}NO_3Na^+$	588.53	$C_{18}H_{34}O_2^+$	282.26
**Cholesteryl suphate**	$C_{27}H_{45}^+$	369.35		
**Cholesterol**	$C_{27}H_{45}^+$	369.35		
	$C_{27}H_{45}O^+$	385.35		
	$C_{37}H_{70}O_5Na^+$	617.51		
**Phosphatidylcholine PC28:0**	$C_8H_{19}NO_4P^+$	224.10	$C_2H_6N^+$	44.05
	$C_{36}H_{73}NO_8P^+$	678.51	$C_5H_{12}N^+$	86.10
			$C_5H_{14}NO^+$	104.11
			$C_2H_6O_4P^+$	125.00
			$C_5H_{13}NO_3P^+$	166.06
			$C_5H_{15}NO_4P^+$	184.07
**Phosphatodylcholine PC32:0**	$C_8H_{19}NO_4P^+$	224.10	$C_2H_6N^+$	44.05
	$C_{40}H_{81}NO_8P^+$	734.57	$C_5H_{12}N^+$	86.10
			$C_5H_{14}NO^+$	104.11
			$C_2H_6O_4P^+$	125.00
			$C_5H_{13}NO_3P^+$	166.06
			$C_5H_{15}NO_4P^+$	184.07
**Phosphatodylcholine PC34:1**	$C_8H_{19}NO_4P^+$	224.10	$C_2H_6N^+$	44.05
	$C_{42}H_{83}NO_8P^+$	760.59	$C_5H_{12}N^+$	86.10
			$C_5H_{14}NO^+$	104.11
			$C_2H_6O_4P^+$	125.00
			$C_5H_{13}NO_3P^+$	166.06
			$C_5H_{15}NO_4P^+$	184.07
**Phosphatodylcholine PC34:2**	$C_8H_{19}NO_4P^+$	224.10	$C_2H_6N^+$	44.05
	$C_{42}H_{81}NO_8P^+$	758.57	$C_5H_{12}N^+$	86.10
			$C_5H_{14}NO^+$	104.11
			$C_2H_6O_4P^+$	125.00
			$C_5H_{13}NO_3P^+$	166.06
			$C_5H_{15}NO_4P^+$	184.07
	C41H82NO8PK$^+$	786.54		
**Sphingomyelin SM34:1**			C2H6N$^+$	44.05
			C5H12N$^+$	86.10
			C5H14NO$^+$	104.11
			C2H6O4P$^+$	125.00
			C5H13NO3P$^+$	166.06
			C5H15NO4P$^+$	184.07
**Alanine**	NH4$^+$	18.03	C3H6NO2$^+$	88.04
	CH4N$^+$	30.03		

	$C2H6N^{+}$	44.05		
	$C3H8NO2^{+}$	90.05		
**Aspartic Acid**	$NH4^{+}$	18.03	$CH4N^{+}$	30.03
	$C2H4NO2^{+}$	74.02	$C2H6N^{+}$	44.05
	$C3H6NO2^{+}$	88.04	$C3H6N^{+}$	56.05
	$C4H8NO4^{+}$	134.04	$C2H6NO^{+}$	60.04
			$C4H10N^{+}$	72.08
**Isoleucine**	NH4+	18.03	$CH4N^{+}$	30.03
	C4H8N+	70.07	$C2H6N^{+}$	44.05
	C2H4NO2+	74.02	$C3H6N^{+}$	56.05
	C5H12N+	86.10	$C4H10N^{+}$	72.08
**Asparagine**	NH4+	18.03	$CH4N^{+}$	30.03
	C2H6NO+	60.04	$C2H6N^{+}$	44.05
	C3H4NO+	70.03	$C3H6N^{+}$	56.05
	C2H4NO2+	74.02	$C4H8N^{+}$	70.07
	C3H7N2O+	87.06	$C5H10N^{+}$	84.08
	C4H9N2O3+	133.07	$C3H6NO2^{+}$	88.04
**Histidine**	NH4+	18.03	$CH4N^{+}$	30.03
	C3H4N2+	68.04	$C2H6N^{+}$	44.05
	C4H5N2+	81.05	$C3H6N^{+}$	56.05
	C4H6N2+	82.06		
	C5H8N3+	110.08		
**Lysine**	NH4+	18.03	$CH4N^{+}$	30.03
	C5H10N+	84.08	$C2H6N^{+}$	44.05
			$C3H6N^{+}$	56.05
			$C4H8N^{+}$	70.07
			$C4H10N^{+}$	72.08
			$C2H4NO2^{+}$	74.02
			$C5H12N^{+}$	86.10
**Methionine**	NH4+	18.03	$CH4N^{+}$	30.03
	H3S+	35.00	$C2H6N^{+}$	44.05
	C2H5S+	61.01	$C3H6N^{+}$	56.05
			$C4H8N^{+}$	70.07
			$C2H4NO2^{+}$	74.02
			$C5H12N^{+}$	86.10
**Ornithine**	NH4+	18.03	$CH4N^{+}$	30.03
			$C2H6N^{+}$	44.05
			$C3H6N^{+}$	56.05
			$C4H8N^{+}$	70.07
			$C2H4NO2^{+}$	74.02

			$C5H12N^{+}$	86.10
**Serine**	NH4+	18.03	$CH4N^{+}$	30.03
	C2H6NO+	60.04	$C2H6N^{+}$	44.05
			$C2H4NO2^{+}$	74.02
			$C5H12N^{+}$	86.10
			$C3H6NO2^{+}$	88.04
**Threonine**	NH4+	18.03	$CH4N^{+}$	30.03
	C2H5O+	45.03	$C2H6N^{+}$	44.05
	C3H6N+	56.05	$C4H8N^{+}$	70.07
	C3H8NO+	74.06	$C5H12N^{+}$	86.10
**Tyrosine**	NH4+	18.03	$CH4N^{+}$	30.03
	C7H7O+	107.05	$C2H6N^{+}$	44.05
	C8H10NO+	136.08	$C3H6N^{+}$	56.05
			$C5H12N^{+}$	86.10
**Valine**	NH4+	18.03	$CH4N^{+}$	30.03
	C4H10N+	72.08	$C2H6N^{+}$	44.05
			$C3H6N^{+}$	56.05
			$C4H8N^{+}$	70.07
			$C5H12N^{+}$	86.10

Table S 4. Calculated monoisotopic mass for lipids detected using SIMS.

Positive

Compound	Adduct	*m/z*
**Choline**	$[M+H]^+$	104.107
**Phosphoryl**	$[[M+H]^+$	125.000
**PC Headgroup**	$[M+H]^+$	184.073
**Cholesterol**	$[M+H]^+$	369.342
**LPC 16:0**	$[M+H]^+$	496.340
	$[M+Na]^+$	518.322
	$[M+K]^+$	534.296
**LPC 18:0**	$[M+H]^+$	524.371
	$[M+Na]^+$	546.353
	$[M+K]^+$	562.327
**SM d34:1**	$[M+H]^+$	703.575
	$[M+Na]^+$	725.557
	$[M+K]^+$	741.531
**PC 32:0**	$[M+H]^+$	734.569
	$[M+Na]^+$	756.551
	$[M+K]^+$	772.525
**PC 34:2**	$[M+H]^+$	758.569
	$[M+Na]^+$	780.551
	$[M+K]^+$	796.525
**SM d42:1**	$[M+H]^+$	815.700
	$[M+Na]^+$	837.682
	$[M+K]^+$	853.656

Negative

Compound	Adduct	*m/z*
**Palmitic Acid (FA(16:0))**	$[M-H]^-$	255.233
**Stearic Acid (FA(18:0))**	$[M-H]^-$	283.264
**Oleic Acid**	$[M-H]^-$	281.249
**Linoleic Acid**	$[M-H]^-$	279.233
**Myristic Acid**	$[M-H]^-$	227.202
**PI head**	$[C_6H_{10}PO_8]^-$	241.040
**Arachidonic acid (FA(20:4))**	$[M-H]^-$	303.233
**Cholesteryl sulfate**	$[M-H]^-$	465.304
**Sapienic Acid (FA(16:1))**	$[M-H]^-$	253.217
**PI(38:4)**	$[M-H]^-$	885.550

Table S 5. Water cluster SIMS experimental parameters.

	Type of Sample	Beam energy (keV)	Cluster size	Beam current (pA)	Pixel resolution	Dose (ions/cm^2)	Shots/pixel	Mass range	Total analysis area (µm)
**Exp. 1**	Liver homogenate	70	30 – 31k	5-8	128 × 128	1.23 – 1.47 $\times 10^{12}$	400	130-1500	1000 × 1000
	Porcine skin	70	28k	8	128 × 128	5.88×10^{12}	296	130-1200	1000 × 1500
**Exp. 2**	Liver homogenate	70	32k	3-4	128 × 128	4.92×10^{12}	992	40-890	500 × 500
	Porcine skin	70	32k	3	128 × 128	4.92×10^{12}	992	40-890	1000 × 1000

Table S 6. Multiple t-test results comparing the TIC normalised area measured from liver tissue homogenates mounted on Si wafer, PET and carbon coated PET (C-PET) and measured using Bi-SIMS in negative ion mode.

	Below threshold?	P value	Mean of Si Wafer	Mean of PET	Difference	SE of difference	t ratio	df	Adjusted P Value
**Palmitic Acid**	No	0.060129	0.006787	0.004933	0.001853	0.0006583	2.815	3.294	0.391101
**Stearic Acid**	No	0.068722	0.004203	0.00364	0.0005633	0.0001612	3.495	2.086	0.392487
**Oleic Acid**	No	0.196766	0.00348	0.00261	0.00087	0.0005294	1.643	3.071	0.583736
**Linoleic Acid**	No	0.307096	0.003443	0.002823	0.00062	0.0005025	1.234	2.927	0.667325
**Myristic Acid**	No	0.332292	0.0006667	0.0004367	0.00023	0.0002087	1.102	4	0.667325
**PI Head**	No	0.051879	0.001003	0.0009133	0.00009	0.00002427	3.709	2.324	0.38088
**Arachidonic Acid**	No	0.149188	0.0009733	0.0008867	0.00008667	0.0000411	2.109	2.371	0.554172
**Cholsteryl Sulphate**	No	0.739562	0.0002233	0.0002367	-0.00001333	0.0000359	0.3714	2.497	0.739562
**Sapienic Acid**	No	0.076086	0.0008367	0.0006333	0.0002033	0.00006289	3.233	2.151	0.392487
	**Below threshold?**	**P value**	**Mean of Si Wafer**	**Mean of C-PET**	**Difference**	**SE of difference**	**t ratio**	**df**	**Adjusted P Value**
**Palmitic Acid**	Yes	0.000324	0.006787	0.001377	0.00541	0.000466	11.61	3.98	0.002909
**Stearic Acid**	Yes	0.007952	0.004203	0.001127	0.003077	0.0002828	10.88	2.027	0.032397
**Oleic Acid**	Yes	0.001427	0.00348	0.0006867	0.002793	0.0003014	9.268	3.477	0.008534
**Linoleic Acid**	Yes	0.0011	0.003443	0.0007733	0.00267	0.0002687	9.935	3.497	0.007676
**Myristic Acid**	No	0.057754	0.0006667	0.00008667	0.00058	0.0001494	3.882	2.057	0.057754
**PI Head**	Yes	0.00805	0.001003	0.00034	0.0006633	0.00007902	8.394	2.379	0.032397
**Arachidonic Acid**	Yes	0.009431	0.0009733	0.00031	0.0006633	0.00007126	9.309	2.117	0.032397
**Cholsteryl Sulphate**	Yes	0.00077	0.0002233	0.00006667	0.0001567	0.000017	9.217	4	0.006142
**Sapienic Acid**	Yes	0.006565	0.0008367	0.0002033	0.0006333	0.00005963	10.62	2.169	0.032397
	**Below threshold?**	**P value**	**Mean of PET**	**Mean of C-PET**	**Difference**	**SE of difference**	**t ratio**	**df**	**Adjusted P Value**
**Palmitic Acid**	No	0.010336	0.004933	0.001377	0.003557	0.0006464	5.502	3.155	0.060433
**Stearic Acid**	Yes	0.003683	0.00364	0.001127	0.002513	0.0003238	7.762	3.162	0.03266
**Oleic Acid**	No	0.041514	0.00261	0.0006867	0.001923	0.000495	3.885	2.504	0.10247
**Linoleic Acid**	No	0.034444	0.002823	0.0007733	0.00205	0.0004745	4.321	2.437	0.10247
**Myristic Acid**	No	0.137974	0.0004367	0.00008667	0.00035	0.0001478	2.367	2.058	0.137974
**PI Head**	No	0.016306	0.0009133	0.00034	0.0005733	0.00007579	7.565	2.031	0.078916
**Arachidonic Acid**	Yes	0.004801	0.0008867	0.00031	0.0005767	0.00008048	7.165	3.14	0.037771
**Cholsteryl Sulphate**	No	0.026665	0.0002367	0.00006667	0.00017	0.0000359	4.735	2.497	0.10247
**Sapienic Acid**	Yes	0.00724	0.0006333	0.0002033	0.00043	0.00008498	5.06	3.988	0.049593

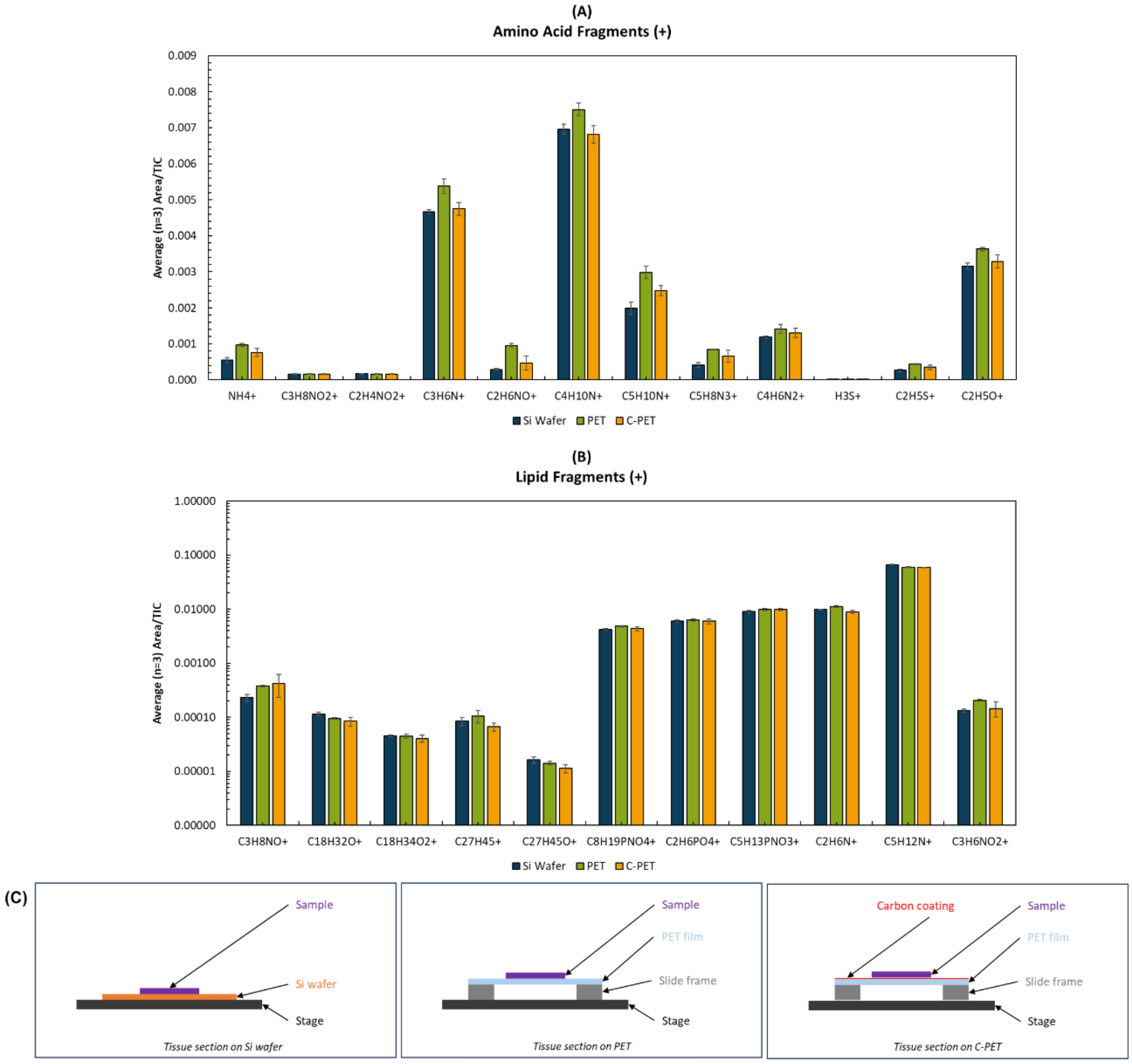

Figure S 2. (A-B) Average (n=3) TIC normalised peak area of tentatively assigned features in negative ion mode measured using bismuth SIMS from liver tissue homogenates mounted on Si wafer, PET and carbon coated PET (C-PET) substrates; (B) Schematic representation (not to scale) of the cross-section view of the sample arrangements described for this experiment.

Table S 7. Kruskal-Wallis test results comparing Si wafer, PET and carbon coated PET (C-PET) as sample substrates in positive ion mode for Bi-SIMS.

**Table Analyzed**	BiSIMS - SiWafer_PET_CPET - Pos
**Kruskal-Wallis test**	
**P value**	0.8846
**Exact or approximate P value?**	Approximate
**P value summary**	ns
**Do the medians vary signif. (P < 0.05)?**	No
**Number of groups**	3
**Kruskal-Wallis statistic**	0.2452
**Data summary**	
**Number of treatments (columns)**	3
**Number of values (total)**	81

**Number of families**	1					
**Number of comparisons per family**	3					
**Alpha**	0.05					
**Dunn's multiple comparisons test**	**Mean rank diff.**	**Significant?**	**Summary**	**Adjusted P Value**		
**Si Wafer vs. PET**	-3.000	No	ns	>0.9999	A-B	
**Si Wafer vs. C-PET**	-0.6111	No	ns	>0.9999	A-C	
**PET vs. C-PET**	2.389	No	ns	>0.9999	B-C	
**Test details**	**Mean rank 1**	**Mean rank 2**	**Mean rank diff.**	**n1**	**n2**	**Z**
**Si Wafer vs. PET**	39.80	42.80	-3.000	27	27	0.4685
**Si Wafer vs. C-PET**	39.80	40.41	-0.6111	27	27	0.09544
**PET vs. C-PET**	42.80	40.41	2.389	27	27	0.3731
**Compact letter display**						
**PET**	A					
**C-PET**	A					
**Si Wafer**	A					

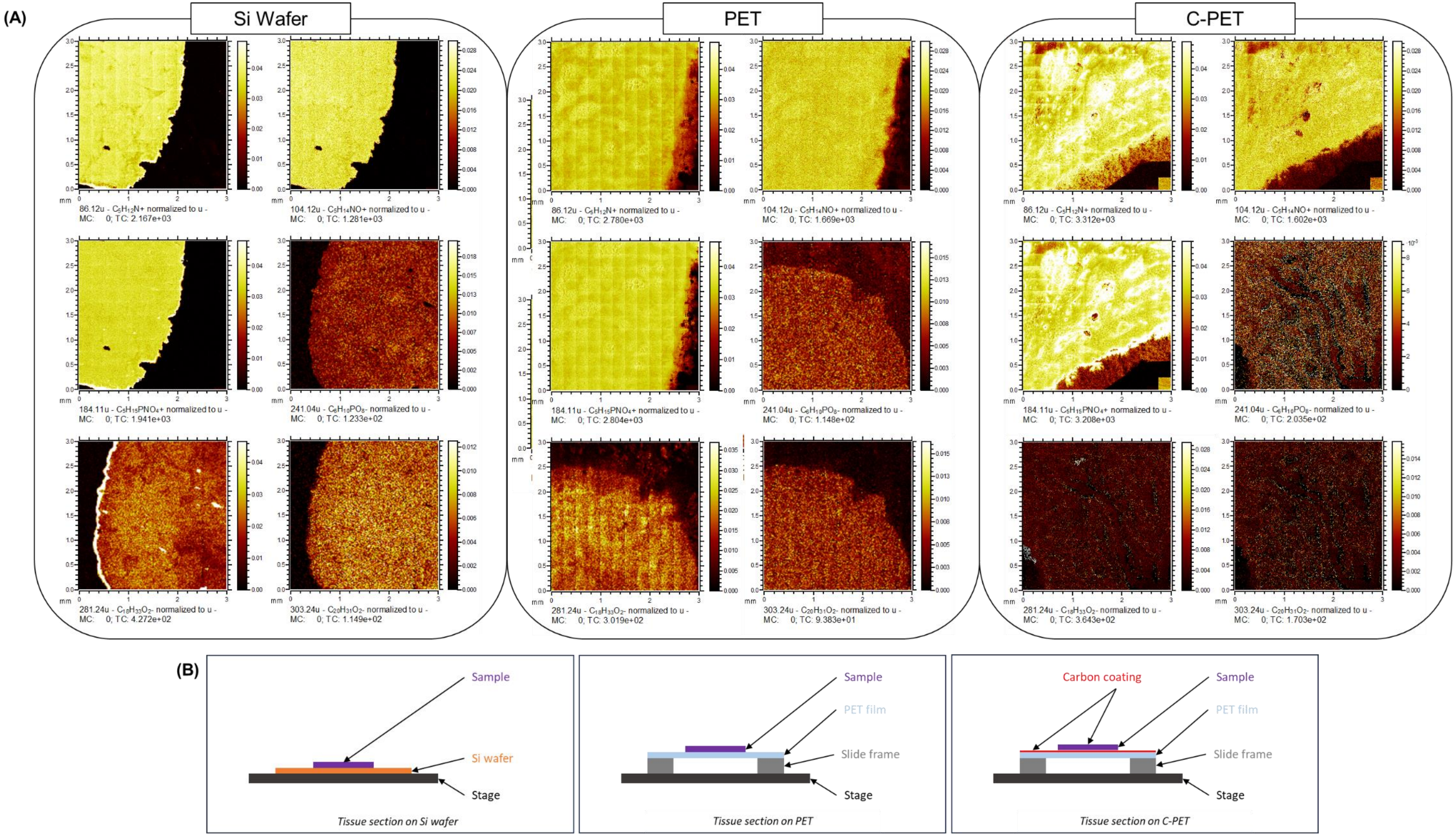

Figure S 3. (A) Bi-SIMS ion maps (3 x 3 mm) measured from liver tissue homogenates mounted on Si wafer (left), PET (middle) and C-PET (right); (B) Schematic representation (not to scale) of the cross-section view of the sample arrangements described for this experiment.

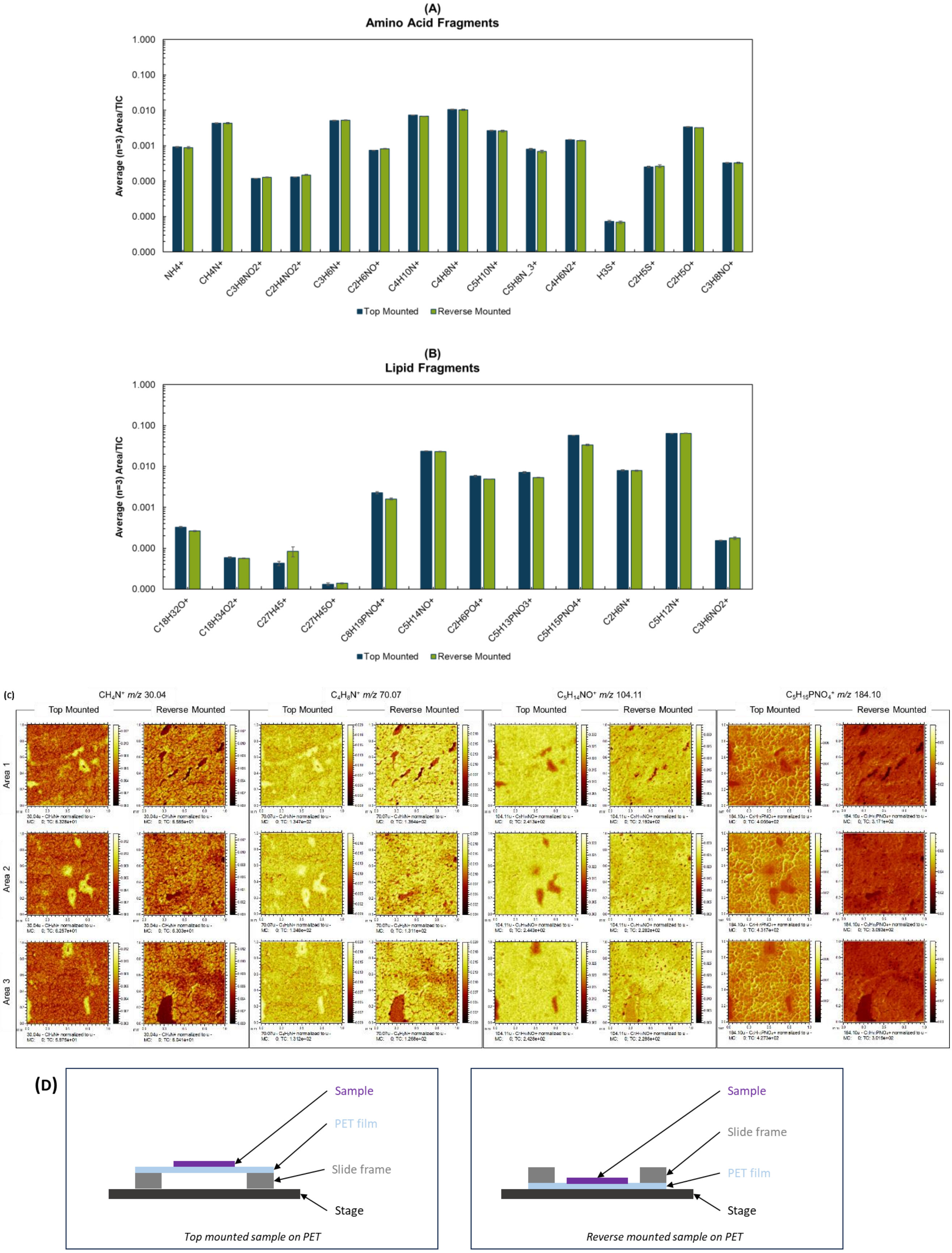

Figure S 4. (A-B) Average (n=3) TIC-normalised peak area of tentatively assigned fragment peaks in positive ion mode measured using Bi-SIMS from liver tissue homogenates measured from top and reverse mounted arrangements; (C) Bi-SIMS ion maps (1 x 1 mm) measured from Areas 1, 2 and 3 on liver tissue homogenates top and reverse mounted on PET

membrane slides and (D) schematic representation (not to scale) of the cross-section view of the sample arrangements described for this experiment.

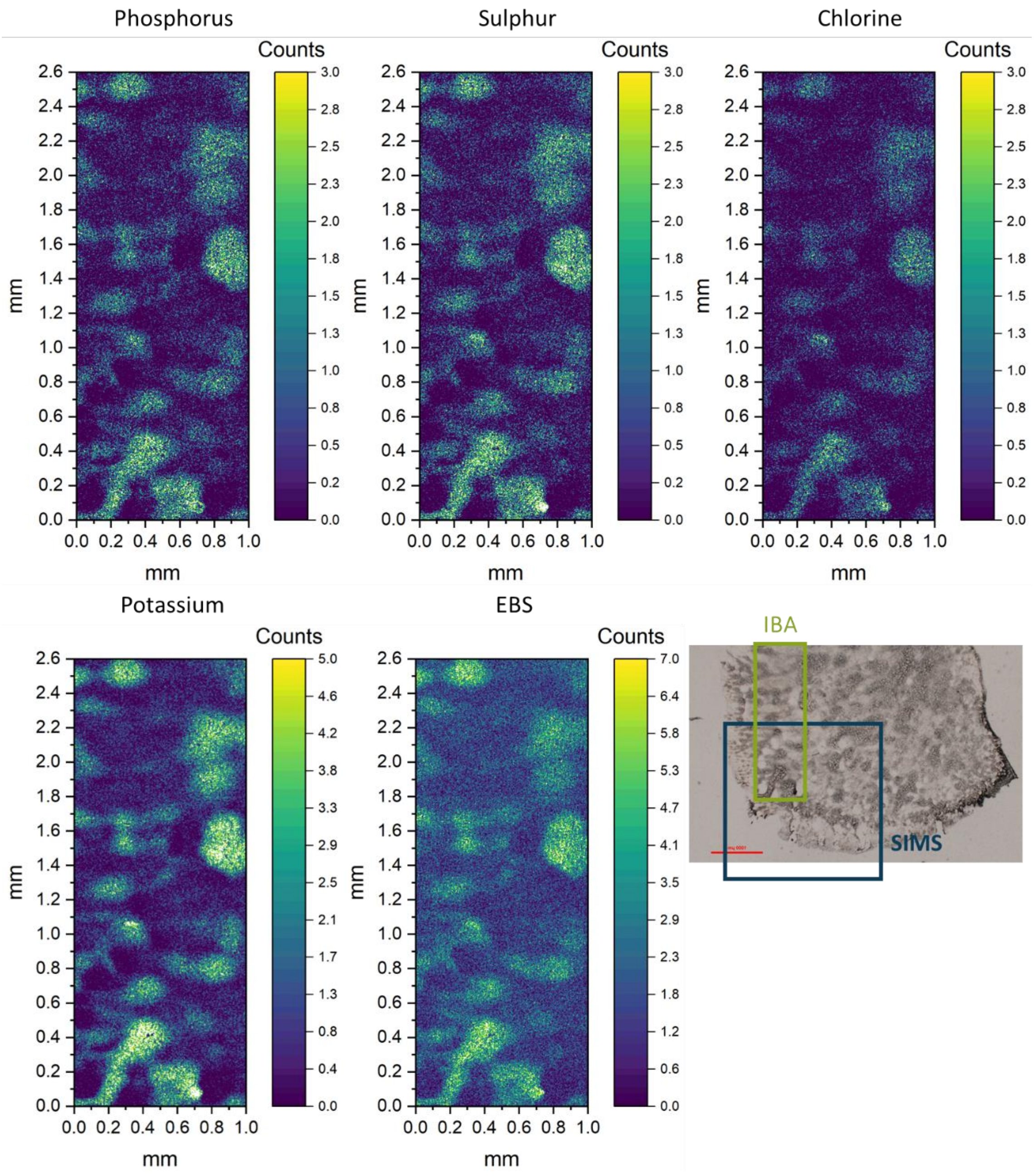

Figure S 5. PIXE and EBS maps of liver tissue homogenates mounted on PET analysed sequentially using Bi-SIMS and ion beam analysis. Images are 256 x 256 pixels in a 1 x 3 array.

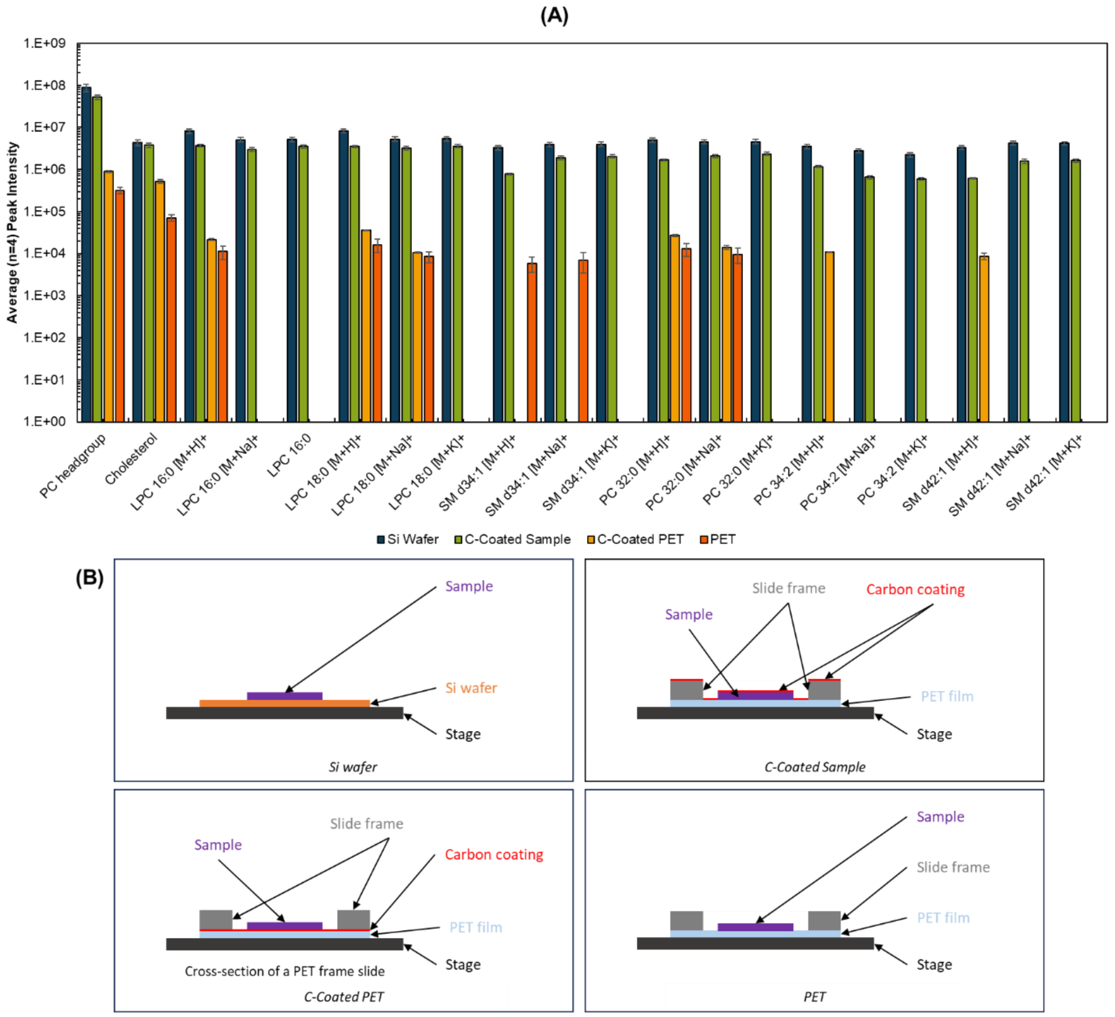

Figure S 6. (A) Average (n=4) TIC-normalised peak intensity of tentatively assigned lipid peaks measured using water cluster SIMS from liver tissue homogenates prepared under different arrangements positive ion mode.

Table S 8. Kruskal-Wallis test results comparing different samples arrangements (Si wafer, C-coated sample, C-coated PET and PET) tested on liver homogenates using water cluster SIMS in positive ion mode.

Table Analyzed	W-SIMS - Si_CCoating_PET - Pos - UnNormalised
Kruskal-Wallis test	
P value	<0.0001
Exact or approximate P value?	Approximate
P value summary	****
Do the medians vary signif. (P < 0.05)?	Yes
Number of groups	4
Kruskal-Wallis statistic	44.25
Data summary	
Number of treatments (columns)	4
Number of values (total)	58

Number of families	1					
Number of comparisons per family	6					
Alpha	0.05					
**Dunn's multiple comparisons test**	**Mean rank diff.**	**Significant?**	**Summary**	**Adjusted P Value**		
Si Wafer vs. C-Coated Sample	15.30	Yes	*	0.0250	A-B	
Si Wafer vs. C-Coated PET	34.38	Yes	****	<0.0001	A-C	
Si Wafer vs. PET	38.27	Yes	****	<0.0001	A-D	
C-Coated Sample vs. C-Coated PET	19.08	Yes	*	0.0292	B-C	
C-Coated Sample vs. PET	22.97	Yes	**	0.0042	B-D	
C-Coated PET vs. PET	3.889	No	ns	>0.9999	C-D	
**Test details**	**Mean rank 1**	**Mean rank 2**	**Mean rank diff.**	**n1**	**n2**	**Z**
Si Wafer vs. C-Coated Sample	46.05	30.75	15.30	20	20	2.865
Si Wafer vs. C-Coated PET	46.05	11.67	34.38	20	9	5.073
Si Wafer vs. PET	46.05	7.778	38.27	20	9	5.646
C-Coated Sample vs. C-Coated PET	30.75	11.67	19.08	20	9	2.815
C-Coated Sample vs. PET	30.75	7.778	22.97	20	9	3.389
C-Coated PET vs. PET	11.67	7.778	3.889	9	9	0.4885
**Compact letter display**						
Si Wafer	A					
C-Coated Sample	A					
C-Coated PET	B					
PET	B					

Table S 9. Multiple t-test results comparing the peak intensities measured from liver tissue homogenates mounted on Si wafer, PET and carbon coated PET (C-PET) using water cluster SIMS.

	Below threshold?	P value	Mean of Si Wafer	Mean of C-Coated Sample	Difference	SE of difference	t ratio	df	Adjusted P Value
**Palmitic Acid (FA(16:0))**	Yes	0.000007	196199265	67492472	128706793	2362618	54.48	3.232	0.000047
**Stearic Acid (FA(18:0))**	Yes	<0.000001	163758701	65819631	97939070	2427248	40.35	4.800	0.000003
**Oleic Acid**	Yes	<0.000001	178348224	48337770	130010454	2913004	44.63	4.490	0.000004
**Linoleic Acid**	Yes	0.000004	173552564	41507533	132045031	3484615	37.89	3.881	0.000031
**Myristic Acid**	Yes	0.000218	5654240	1281669	4372571	212266	20.60	3.066	0.001088
**PI head**			18300602						
**Arachidonic acid (FA(20:4))**	Yes	0.000134	54462324	13645665	40816659	1941447	21.02	3.267	0.000803
**Cholesteryl sulfate**	Yes	0.000726	1498522	501381	997141	87688	11.37	3.466	0.001088
**Sapienic Acid (FA(16:1))**	Yes	0.000331	51416978	8408468	43008510	2316222	18.57	3.014	0.001088
**PI(38:4)**	Yes	0.000497	13053028	4231152	8821876	559570	15.77	3.060	0.001088
	**Below threshold?**	**P value**	**Mean of Si Wafer**	**Mean of C-Coated PET**	**Difference**	**SE of difference**	**t ratio**	**df**	**Adjusted P Value**
**Palmitic Acid (FA(16:0))**	Yes	0.000003	196199265	3775026	192424239	2330740	82.56	3.065	0.000028
**Stearic Acid (FA(18:0))**	Yes	0.000005	163758701	1117365	162641336	2103128	77.33	3.006	0.000037
**Oleic Acid**	Yes	0.000007	178348224	2435867	175912357	2589800	67.93	3.004	0.000049
**Linoleic Acid**	Yes	0.000015	173552564	2411718	171140846	3249515	52.67	3.001	0.000090
**Myristic Acid**	Yes	0.000121	5654240	131461	5522779	211219	26.15	3.006	0.000389
**PI head**			18300602						
**Arachidonic acid (FA(20:4))**	Yes	0.000097	54462324	789077	53673247	1899726	28.25	3.001	0.000389
**Cholesteryl sulfate**			1498522						
**Sapienic Acid (FA(16:1))**	Yes	0.000207	51416978	601895	50815083	2313572	21.96	3.000	0.000389
**PI(38:4)**	Yes	0.000171	13053028	13880	13039148	556811	23.42	3.000	0.000389
	**Below threshold?**	**P value**	**Mean of Si Wafer**	**Mean of PET**	**Difference**	**SE of difference**	**t ratio**	**df**	**Adjusted P Value**
**Palmitic Acid (FA(16:0))**	Yes	0.000004	196199265	134334	196064931	2318290	84.57	3.000	0.000029
**Stearic Acid (FA(18:0))**	Yes	0.000005	163758701	48817	163709884	2102053	77.88	3.000	0.000033
**Oleic Acid**	Yes	0.000007	178348224	83300	178264924	2589068	68.85	3.000	0.000040
**Linoleic Acid**	Yes	0.000014	173552564	87060	173465504	3249183	53.39	3.000	0.000072
**Myristic Acid**	Yes	0.000115	5654240	5867	5648373	211111	26.76	3.000	0.000280
**PI head**			18300602						
**Arachidonic acid (FA(20:4))**	Yes	0.000093	54462324	22711	54439613	1899643	28.66	3.000	0.000280
**Cholesteryl sulfate**			1498522						
**Sapienic Acid (FA(16:1))**	Yes	0.000200	51416978	23858	51393120	2313540	22.21	3.000	0.000280
**PI(38:4)**			13053028						
	**Below threshold?**	**P value**	**Mean of C-Coated Sample**	**Mean of C-Coated PET**	**Difference**	**SE of difference**	**t ratio**	**df**	**Adjusted P Value**
**Palmitic Acid (FA(16:0))**	Yes	<0.000001	67492472	3775026	63717446	515570	123.6	4.556	<0.000001
**Stearic Acid (FA(18:0))**	Yes	0.000014	65819631	1117365	64702266	1215569	53.23	3.019	0.000055
**Oleic Acid**	Yes	0.000052	48337770	2435867	45901903	1336731	34.34	3.014	0.000157
**Linoleic Acid**	Yes	0.000072	41507533	2411718	39095815	1260332	31.02	3.010	0.000157
**Myristic Acid**	Yes	0.000003	1281669	131461	1150208	23135	49.72	3.558	0.000024
**PI head**									
**Arachidonic acid (FA(20:4))**	Yes	0.000065	13645665	789077	12856588	401185	32.05	3.013	0.000157
**Cholesteryl sulfate**			501381						
**Sapienic Acid (FA(16:1))**	Yes	0.000005	8408468	601895	7806573	112231	69.56	3.079	0.000030
**PI(38:4)**	Yes	0.000005	4231152	13880	4217272	55508	75.98	3.000	0.000030

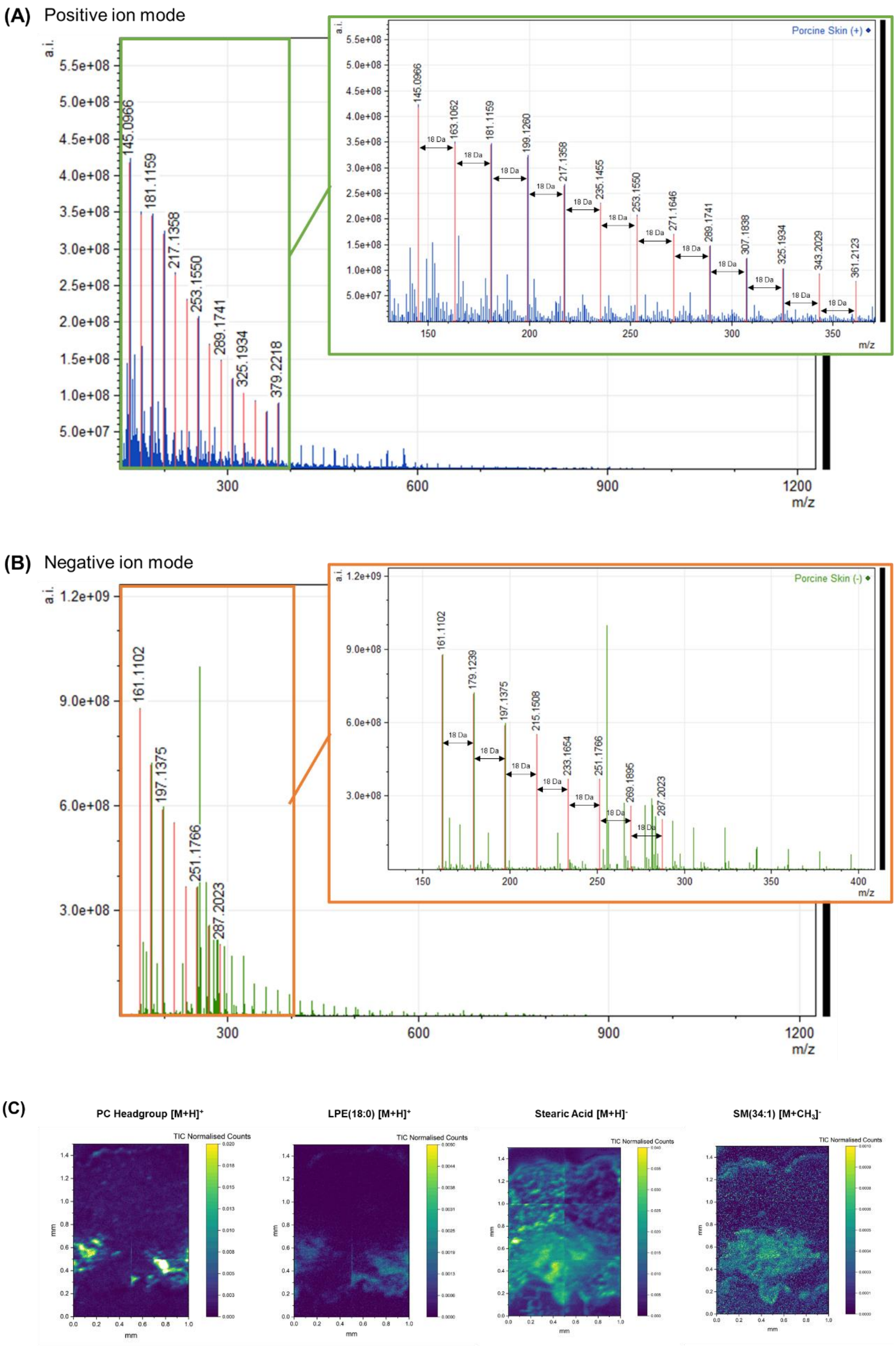

Figure S 7. Total ion spectra for (A) positive and (B) negative ion modes showing the repeating 18 Da units originating from the backscattered water clusters; (C) ion maps for a selection of lipid-related peaks obtained from a carbon-coated porcine skin section, mounted on a PET membrane and analysed using water cluster SIMS.

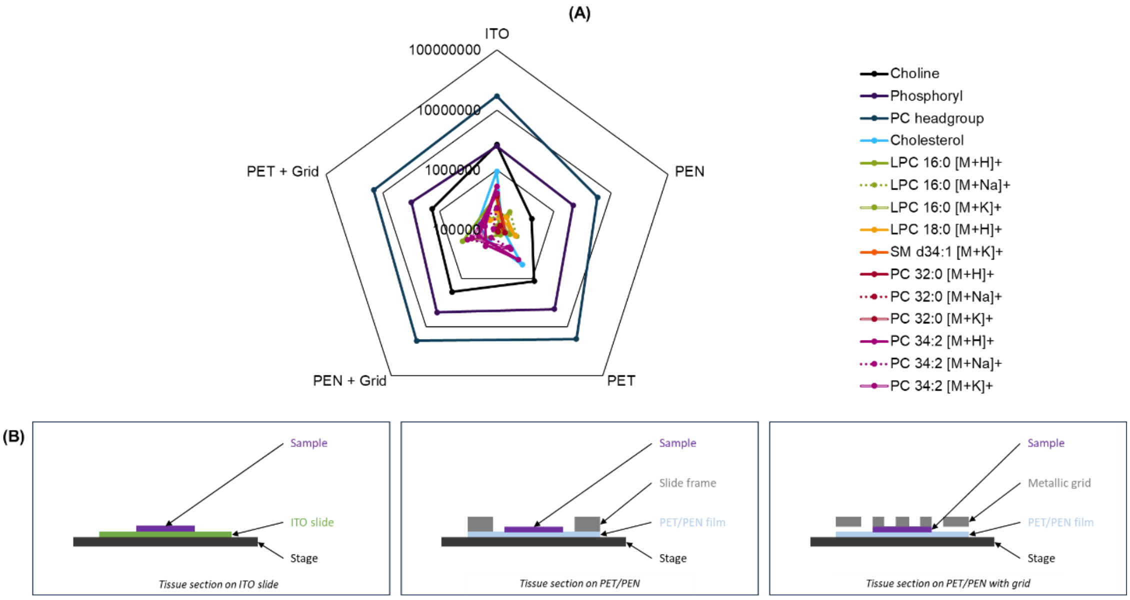

Figure S 8. (A) Average (n=3) peak intensity of tentatively assigned lipid peaks measured using water cluster SIMS from liver tissue homogenates prepared under different arrangements in positive ion mode; (B) schematic representation (not to scale) of the cross-section view of the sample arrangements described for this experiment.

Table S 10. Mann Whitney tests results carried out between PEN + Grid and ITO, PET + Grid and ITO and PEN + Grid and PET + Grid datasets obtained from liver tissue homogenates and measured using water cluster SIMS in negative ion mode.

Table Analyzed	W-SIMS - ITO_PEN_PET - Neg - UnNormalised
Column D	PEN + Grid
vs.	vs.
Column A	ITO
Mann Whitney test	
P value	0.8874
Exact or approximate P value?	Exact
P value summary	ns
Significantly different (P < 0.05)?	No
One- or two-tailed P value?	Two-tailed
Sum of ranks in column A,D	153 , 147
Mann-Whitney U	69
Difference between medians	
Median of column A	2413895, n=12
Median of column D	2511949, n=12
Difference: Actual	98054
Difference: Hodges-Lehmann	-258214

Table Analyzed	W-SIMS - ITO_PEN_PET - Neg - UnNormalised
Column E	PET + Grid
vs.	vs.
Column A	ITO
Mann Whitney test	
P value	0.7553
Exact or approximate P value?	Exact
P value summary	ns
Significantly different (P < 0.05)?	No
One- or two-tailed P value?	Two-tailed
Sum of ranks in column A,E	156 , 144
Mann-Whitney U	66
Difference between medians	
Median of column A	2413895, n=12
Median of column E	2552113, n=12
Difference: Actual	138218
Difference: Hodges-Lehmann	-405846

Table Analyzed	W-SIMS - ITO_PEN_PET - Neg - UnNormalised
Column E	PET + Grid
vs.	vs.
Column D	PEN + Grid
Mann Whitney test	
P value	0.7987
Exact or approximate P value?	Exact
P value summary	ns
Significantly different (P < 0.05)?	No
One- or two-tailed P value?	Two-tailed
Sum of ranks in column D,E	155 , 145
Mann-Whitney U	67
Difference between medians	
Median of column D	2511949, n=12
Median of column E	2552113, n=12
Difference: Actual	40165
Difference: Hodges-Lehmann	-127990

Table S 11. Kruskal-Wallis test results comparing different samples arrangements (ITO, PEN, PET, PEN + Grid and PET + Grid) tested on liver homogenates using water cluster SIMS in positive ion mode.

Table Analyzed	W-SIMS - ITO_PEN_PET - Pos - UnNormalised
Kruskal-Wallis test	
P value	0.3108
Exact or approximate P value?	Approximate
P value summary	ns
Do the medians vary signif. (P < 0.05)?	No
Number of groups	5
Kruskal-Wallis statistic	4.778
Data summary	
Number of treatments (columns)	5
Number of values (total)	68

Number of families	1					
Number of comparisons per family	10					
Alpha	0.05					
Dunn's multiple comparisons test	Mean rank diff.	Significant?	Summary	Adjusted P Value		
ITO vs. PEN	13.83	No	ns	0.9466	A-B	
ITO vs. PET	5.986	No	ns	>0.9999	A-C	
ITO vs. PEN + Grid	10.70	No	ns	>0.9999	A-D	
ITO vs. PET + Grid	12.01	No	ns	0.8828	A-E	
PEN vs. PET	-7.839	No	ns	>0.9999	B-C	
PEN vs. PEN + Grid	-3.125	No	ns	>0.9999	B-D	
PEN vs. PET + Grid	-1.817	No	ns	>0.9999	B-E	
PET vs. PEN + Grid	4.714	No	ns	>0.9999	C-D	
PET vs. PET + Grid	6.022	No	ns	>0.9999	C-E	
PEN + Grid vs. PET + Grid	1.308	No	ns	>0.9999	D-E	
Test details	Mean rank 1	Mean rank 2	Mean rank diff.	n1	n2	Z
ITO vs. PEN	41.70	27.88	13.83	20	8	1.671
ITO vs. PET	41.70	35.71	5.986	20	14	0.8687
ITO vs. PEN + Grid	41.70	31.00	10.70	20	13	1.519
ITO vs. PET + Grid	41.70	29.69	12.01	20	13	1.705
PEN vs. PET	27.88	35.71	-7.839	8	14	0.8945
PEN vs. PEN + Grid	27.88	31.00	-3.125	8	13	0.3517
PEN vs. PET + Grid	27.88	29.69	-1.817	8	13	0.2045
PET vs. PEN + Grid	35.71	31.00	4.714	14	13	0.6190
PET vs. PET + Grid	35.71	29.69	6.022	14	13	0.7907
PEN + Grid vs. PET + Grid	31.00	29.69	1.308	13	13	0.1686
Compact letter display						
PEN + Grid	A					
PET	A					
PET + Grid	A					
ITO	A					
PEN	A					

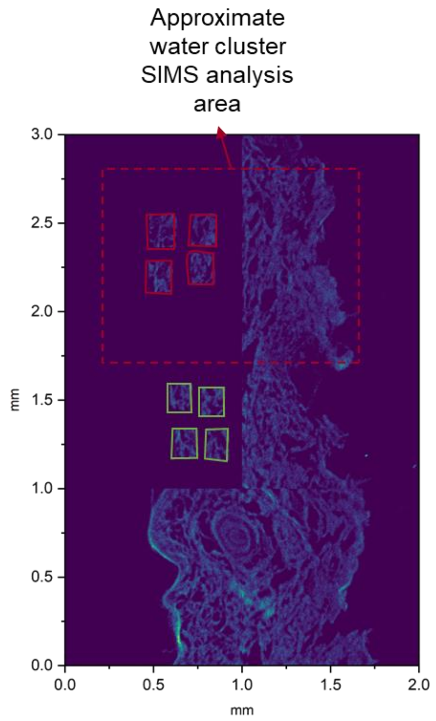

Figure S 9. Regions of interest (ROI) selected from an area previously analysed by water cluster SIMS and a pristine (no previous measurement) area.

Table S 12. Multiple t-test results comparing the elemental concentrations measured using PIXE from an area that had previously been analysed with SIMS and a pristine (no SIMS) area.

	Below threshold?	P value	Mean of SIMS	Mean of No SIMS	Difference	SE of difference	t ratio	df	Adjusted P Value
**P**	No	0.240786	12.40	15.80	-3.400	2.566	1.325	5.156	0.562385
**S**	No	0.145525	29.70	37.20	-7.500	4.469	1.678	5.854	0.466915
**Cl**	No	0.263939	79.50	99.60	-20.10	16.23	1.239	5.711	0.562385
**K**	No	0.327149	3.800	4.800	-1.000	0.9301	1.075	5.503	0.562385

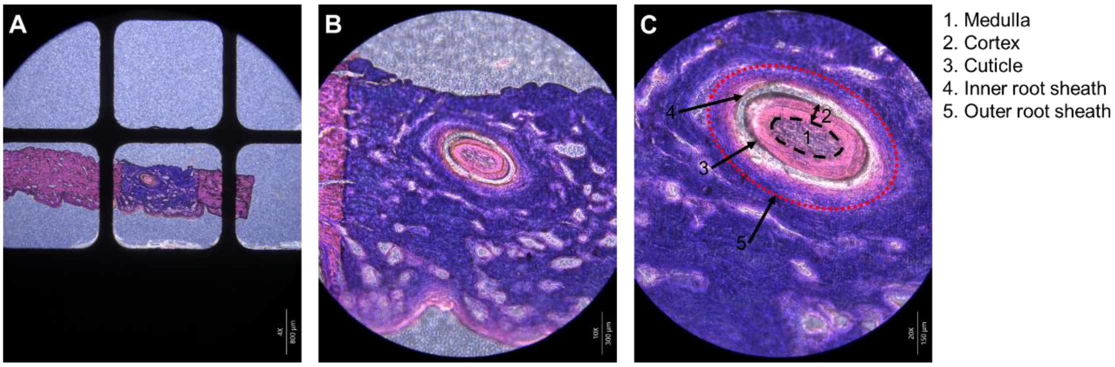

Figure S 10. Porcine skin sample mounted on PEN with a metallic grid on top (as shown in Figure S1(B)). H&E staining was performed after sequential water cluster SIMS and IBA. Optical images taken at (A) 4x, (B) 10x and (C) 20 x magnification. (C) also shows the annotated regions of the hair follicle.

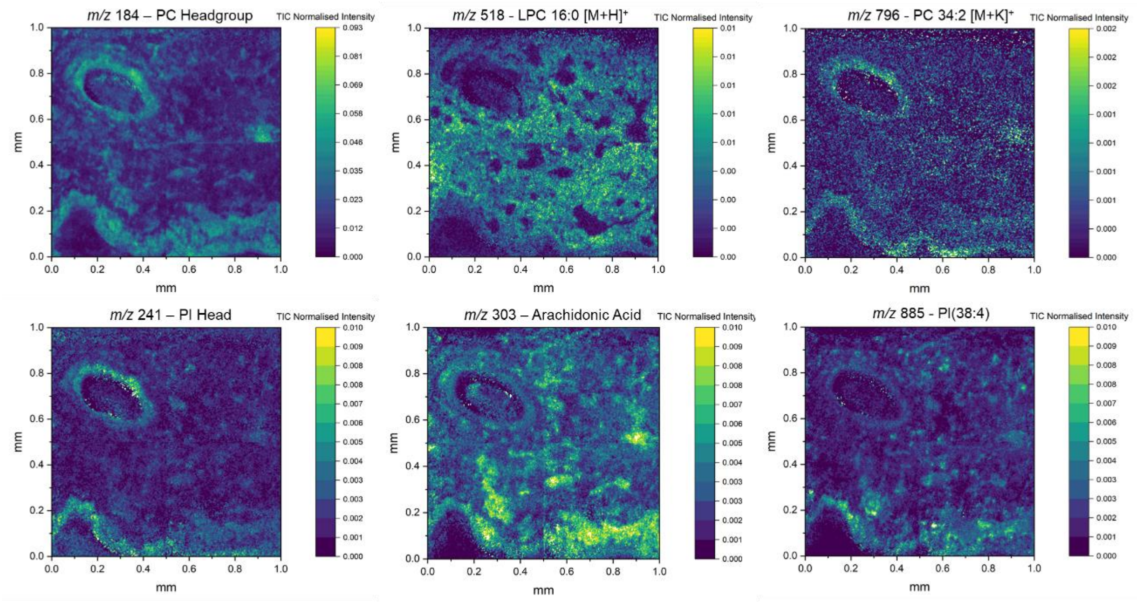

Figure S 11. Ion maps taken from a porcine skin sample mounted on PEN with a metallic grid on top and analysed using water cluster SIMS in positive (top row) and negative (bottom row) ion modes.

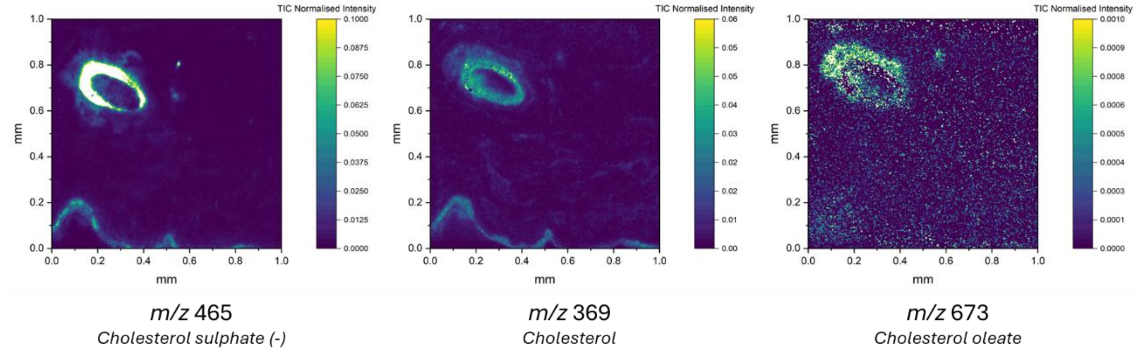

Figure S 12. Ion maps for cholesterol-derived peaks taken from a porcine skin sample mounted on PEN with a metallic grid on top and analysed using water cluster SIMS.
